# TrichomeLess Regulator 3 is required for trichome initial and cuticle biosynthesis in *Artemisia annua*

**DOI:** 10.1186/s43897-024-00085-4

**Published:** 2024-03-19

**Authors:** Boran Dong, Zihan Xu, Xingxing Wang, JinXing Li, Ying Xiao, Doudou Huang, Zongyou Lv, Wansheng Chen

**Affiliations:** 1 Research and Development Center of Chinese Medicine Resources and Biotechnology, Shanghai University of Traditional Chinese Medicine, Shanghai, 201203 China; 2 Department of Pharmacy, Changzheng Hospital, Second Military Medical University, Shanghai, 200003 China

**Keywords:** *Artemisia annua*, Artemisinin, Glandular trichomes, Trichome morphology, Cuticle, Negative regulation

## Abstract

Artemisinin is primarily synthesized and stored in the subepidermal space of the glandular trichomes of *Artemisia annua*. The augmentation of trichome density has been demonstrated to enhance artemisinin yield. However, existing literature lacks insights into the correlation between the stratum corneum and trichomes. This study aims to unravel the involvement of *TrichomeLess Regulator* 3 (*TLR3*), which encodes the transcription factor, in artemisinin biosynthesis and its potential association with the stratum corneum. *TLR3* was identified as a candidate gene through transcriptome analysis. The role of *TLR3* in trichome development and morphology was investigated using yeast two-hybrid, pull-down analysis, and RNA electrophoresis mobility assay. Our research revealed that *TLR3* negatively regulates trichome development. It modulates the morphology of *Arabidopsis thaliana* trichomes by inhibiting branching and inducing the formation of abnormal trichomes in *Artemisia annua*. Overexpression of the *TLR3* gene disrupts the arrangement of the stratum corneum and reduces artemisinin content. Simultaneously, *TLR3* possesses the capacity to regulate stratum corneum development and trichome follicle morphology by interacting with TRICHOME AND ARTEMISININ REGULATOR 1, and CycTL. Consequently, our findings underscore the pivotal role of *TLR3* in the development of glandular trichomes and stratum corneum biosynthesis, thereby influencing the morphology of *Artemisia annua* trichomes.

**Supplementary Information:**

The online version contains supplementary material available at 10.1186/s43897-024-00085-4.

## Core

*TLR3* regulates trichome development and morphology, negatively regulates trichome density and the biosynthesis of Artemisinin, and affects the biosynthesis of cuticles in *A.annua*. The protein ECT2 involving in methylation may regulates the morphology of trichome by binding *TLR3* mRNA.

## Gene & accession numbers

Sequence data from this article can be found in the GenPept/EMBL database under the following accession numbers: TLR3 (PWA99549), CycTL(PWA56939), ADS (AF138959), CYP71AV1 (DQ453967), DBR2 (EU704257), ALDH1 (FJ809784), NFY1 (PWA69343), ECT2 (PWA54528).

## Introduction

Artemisinin, derived from the traditional Chinese herb sweet wormwood (*Artemisia annua* L) of the Asteraceae family, is a highly oxygenated sesquiterpene featuring a distinctive 1,2,4-trioxane ring structure. Artemisinin is considered the primary active compound in artemisinin-based combination therapy (ACT) for the treatment of malaria (Owoloye et al. 2021). Moreover, artemisinin exhibits protective effects against *Mycobacterium tuberculosis* (Zheng et al. 2017), diabetes (Li et al. 2017) and cancer (Carqueijeiro et al. 2020). Although genetically modified budding yeast (*Saccharomyces cerevisiae*) has been engineered to produce artemisinic acid, the precursor of artemisinin (Paddon et al. 2013), plants continue to serve as the primary source (Tang et al. 2014).

The artemisinin biosynthetic pathway has been elucidated in recent years. The precursor, farnesyl diphosphate (FPP) undergoes sequential catalysis to convert into dihydroartemisinic acid (DHAA). This process involves enzymes such as amorpha-4,11-diene synthase (ADS), amorphadiene 12-hydroxylase (the cytochrome P450 71AV1 [CYP71AV1]), artemisinic aldehydeΔ11(13) reductase (double bond reductase 2 [DBR2]) and aldehyde dehydrogenase 1 (ALDH1) (Picaud et al. 2006; Zhang et al. 2008; Teoh et al. 2009; Rydén et al. 2010). Notably, the final steps, from DHAA to artemisinin, occur through nonenzymatic means in vivo (Czechowski et al. 2016).

Genes responsible for components of the artemisinin biosynthetic pathway in *A. annua* are predominantly expressed in glandular trichomes, where artemisinin is synthesized and stored (Olsson et al. 2009). Numerous studies have suggested that increasing the number of trichomes can significantly boost the yield of artemisinin in plants (Xie et al. 2021; Guo et al. 2022; Lv et al. 2022; Chen et al. 2023; Liu et al. 2023). Consequently, understanding the developmental mechanisms governing trichomes holds the potential to enhance artemisinin production.

MYB-type transcription factors (TFs) have been identified as positive regulators of trichome development. However, negative regulation of trichome development has also been documented (Szymanski et al. 1998; Szymanski and Marks 1998; Schellmann et al. 2002) which may hold greater significance. Mutations in several trichome negative regulators have been found to substantially increase trichome number by up to 12 times (Kirik et al. 2004a, b). Certainly, in double mutants of the MYB TF genes *EVOLUTIONARILY CONSERVED C-TERMINAL REGION 1* (*ETC1*) and *TRIPTYCHON* (*TRY*) in *Arabidopsis,* the trichome number per leaf escalates significantly from 27 to 408 (Kirik et al. 2004a, b). MYB TFs are known to interact with other proteins to modulate trichome initiation. In the model plant *Arabidopsis* (*Arabidopsis thaliana*), the MYB TF *GLABRA1* (*GL1*) forms a complex with the basic helix-loop-helix (bHLH) TF *GL3* (also known as ENHANCER OF GL3 [EGL3]) and the WD40 protein TRANSPARENT TESTA GLABRA1 (TTG1) to activate *GL2* transcription (Ishida et al. 2008). Several negative regulators within the MYB TF family, including *TRY*, *CAPRICE* (*CPC*), *ETC1* and *ETC2*, have been identified to influence trichome development (Szymanski and Marks 1998; Schellmann et al. 2002; Kirik et al. 2004a, b; Kirik et al. 2004a, b). In cucumber (*Cucumis sativus*), the MYB TF *CsMYB6* has been identified as a negative regulator of trichome development (Yang et al. 2018). In *A. annua*, AaMIXTA1 interacts with the homeodomain (HD) leucine zipper (ZIP) TF *AaHD8* to positively regulate trichome development (Yan et al. 2017, 2018; Shi et al. 2018) Conversely, certain MYB TFs,, such as *AaMYB5* and *AaMYB16*, have been found to negatively regulate trichome development (Xie et al. 2021). Additionally, the MYB TF *TrichomeLess Regulator 1* (*TLR1*) forms a complex with *WUSCHEL-RELATED HOMEOBOX 1* (*AaWOX1*) and *TLR2* to regulate trichome development; RNA interference (RNAi) of *TLR1* has been shown to increase trichome density by up to 100% (Lv et al. 2022). Consequently, MYB TFs appear to play a conserved role in trichome development. Identifying these MYB TFs and unraveling their specific roles could prove to be a crucial strategy to enhance trichome density, potentially boosting artemisinin production. A deeper comprehension of trichome structure would also contribute to this goal.

Each glandular trichome in *A. annua* is comprised of ten cells, including two stalk cells, three pairs of secretory cells, and two basal cells. The structure is crowned by a bilobed sac covered in cuticle, which holds a pivotal role in trichome development (Duke and Paul 1993). The cuticle layer is formed by a combination of cutin and cuticular wax (Lee and Suh 2022), both of which exert an influence on trichome development.

“Transcription factors, including MYB TFs, have been discovered to play a role in regulating cuticle wax accumulation and trichome development in various plant species, such as tomato (*Solanum lycopersicum*), peach (*Prunus persica*) and *Arabidopsis* (Oshima et al. 2013; Oshima and Mitsuda 2014; Xiong et al. 2020; Yang et al. 2022). In *A. annua*, the cyclin protein known as cyclin trichomeless (CycTL) may influence wax accumulation, potentially inhibiting the development of trichome initial cell development (Dong et al. 2021). Additionally, the suppression of *TAR1* transcript levels through RNAi has been observed to lead to changes in the composition of cuticle wax and alterations in trichome phenotypes (Tan et al. 2015). The MYB TF *AaMIXTA1* has the capacity to increase trichome density by facilitating the accumulation of cuticular wax through the activation of genes responsible for cuticular wax production in *A. annua* (Shi et al. 2018). Consequently, it appears that there is a probable connection between cuticular wax formation and trichome development.

Here, we present compelling evidence indicating that *TLR3* exerts a negative influence on trichome development in both *Arabidopsis* and *A. annua*. Furthermore, we demonstrate that *TLR3* is expressed at various stage of callus development, where it inhibits the formation of trichome initial cells. Lastly, we provide substantiated proof that *TLR3* plays a role in regulating cuticle biosynthesis and impacts trichome morphology by interacting with *CycTL*. Our findings strongly suggest that *TLR3* plays a significant role in cuticle biosynthesis and modulates trichome morphology, ultimately influencing the production of artemisinin in *A. annua*.

## Results

### Screening transcriptomes of different leaves identified a likely trichome-regulatory MYB TF gene

**Fig. 1:**
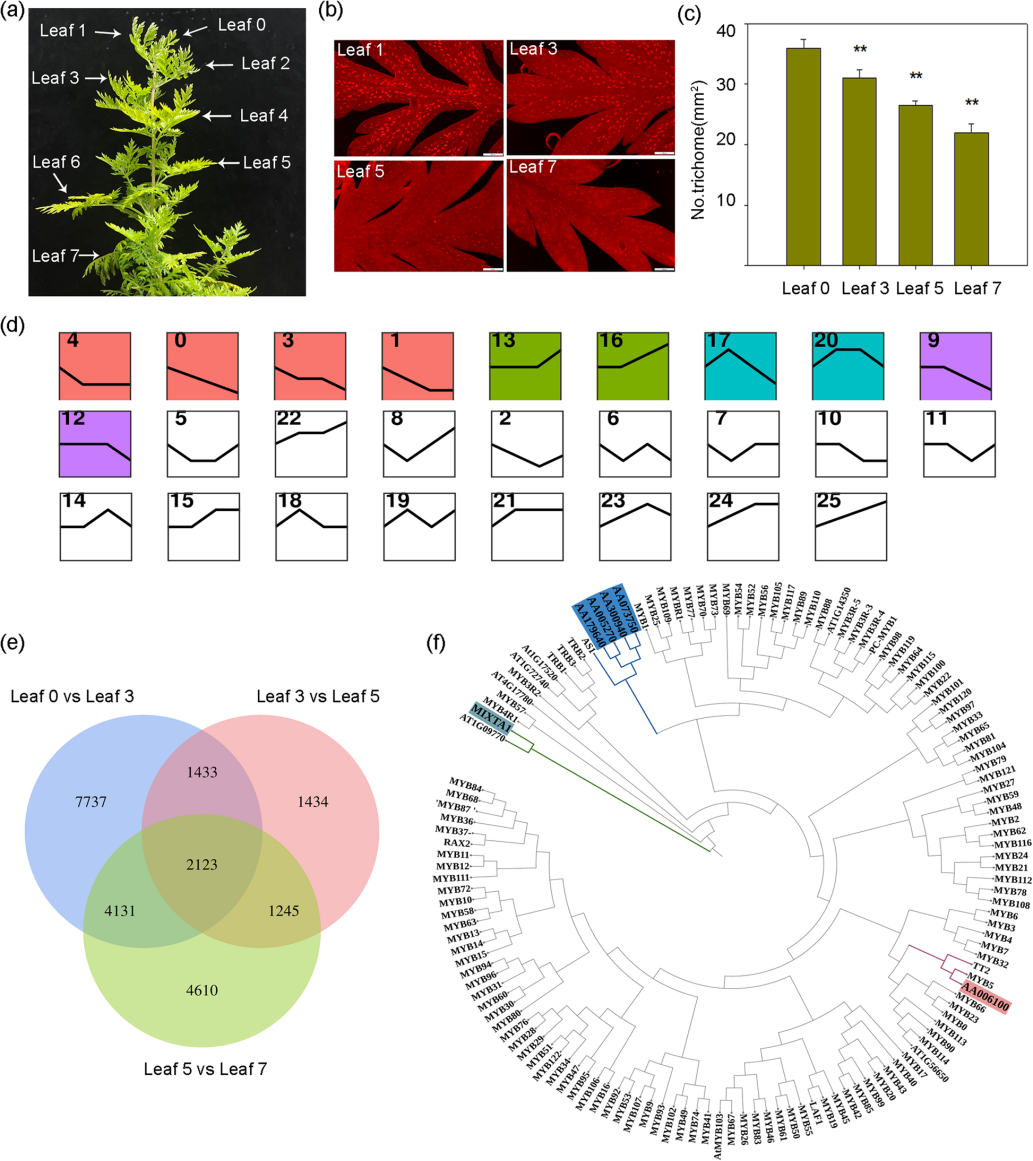
Transcriptome sequencing and differential gene analysis of leaves 0, 3, 5 and 7 of *A*. *annua* plants. **a** Leaves from a three-month-old plant, numbered from top (youngest) to bottom (oldest). **b** Glandular trichomes on leaf 0, leaf 3, leaf 5 and leaf 7 of *A*. *annua* visualized by fluorescence microscopy. Autofluorescence of glandular trichomes was captured with λ_ex_ = 480 nm and λ_em_ = 535 nm. **c** Trichome density in *A*. *annua* leaf 0, leaf 3, leaf 5 and leaf 7. Asterisks indicate significant differences relative to leaf 0 (Student’s *t*-test; **, *P* < 0.01). Data are means ± standard error (SE). **d** Clustering analysis of 2,123 genes in different leaves. The *x* axis represents leave samples from four developmental stages (leaf 0, leaf 3, leaf 5 and leaf 7); the *y* axis represents gene expression. Twenty-five clusters were identified. **e** Venn diagram showing the extent of overlap between differentially expressed genes in the comparisons leaf 0 vs leaf 3; leaf 3 vs leaf 5; and leaf 5 vs leaf 7. **f** Maximum likelihood phylogenetic tree reconstructed based on six identified *A*. *annua* transcription factors and 131 *Arabidopsis thaliana* transcription factors

Earlier investigations have revealed that genes associated with artemisinin biosynthesis, including *ADS*, *CYP71AV1*, *DBR2*, and *ALDH1*, exhibit predominant expression in glandular trichomes. These trichomes are predominantly found in younger *A. annua* leaves, with higher densities compared to older leaves. (Olofsson et al. 2011) (Fig. 1a–c). We postulated that a regulator of artemisinin biosynthesis would demonstrate a similar expression profile to genes associated with trichome development. Consequently, we conducted a comprehensive transcriptome deep sequencing (RNA-seq) analysis of leaves at various developmental stages in *A. annua*. Specifically, we harvested the youngest leaf (leaf 0) from three-month-old plant seedlings, along with leaves 3, 5, and 7. After aligning sequencing reads to the *A. annua* reference genome (PRJCA009719), we detected genes displaying distinct expression patterns between leaf 0 and leaves 3, 3 and 5, and between leaf 5 and leaf 7. A total of 2,123 genes exhibited significant differential expression across all three comparisons, implying their potential involvement in trichome development (Fig. 1e). Employing these genes for hierarchical clustering analysis resulted in 25 clusters (Fig. 1d). Our focus centered on genes within colored clusters exhibiting significant expression trends (*p* < 0.05). Within these 10 clusters, we identified 41 MYB TF genes for further investigation, as MYB family members have demonstrated substantial contributions to trichome development in other plant species.

We employed the QuickGO tool (https://www.ebi.ac.uk/QuickGO) for functional prediction of all 41 MYB TFs, revealing that six genes (AA005270, AA006100, AA179640, AA366500, AA300940 and AA0737504) might be associated with trichome development (Table S2). Notably, AA366500 corresponds to *MIXTA1* (Shi et al. 2018), a gene previously implicated in modulating trichome development in *A. annua.* This led us to hypothesize that the remaining five genes may also play a role in regulating this developmental program. To refine our candidates, we constructed a phylogenetic tree using the six *A. annua* TF genes and all MYB TF genes from *Arabidopsis* (Fig. 1f). Our analysis revealed that the protein encoded by AA006100 clusters with *Arabidopsis MYB5*, a known regulator of trichome development. Consequently, we selected AA006100 as a candidate gene, naming it *TLR3* (*Trichomeless Regulator 3*).

### Characterization of TLR3 expression

**Fig. 2:**
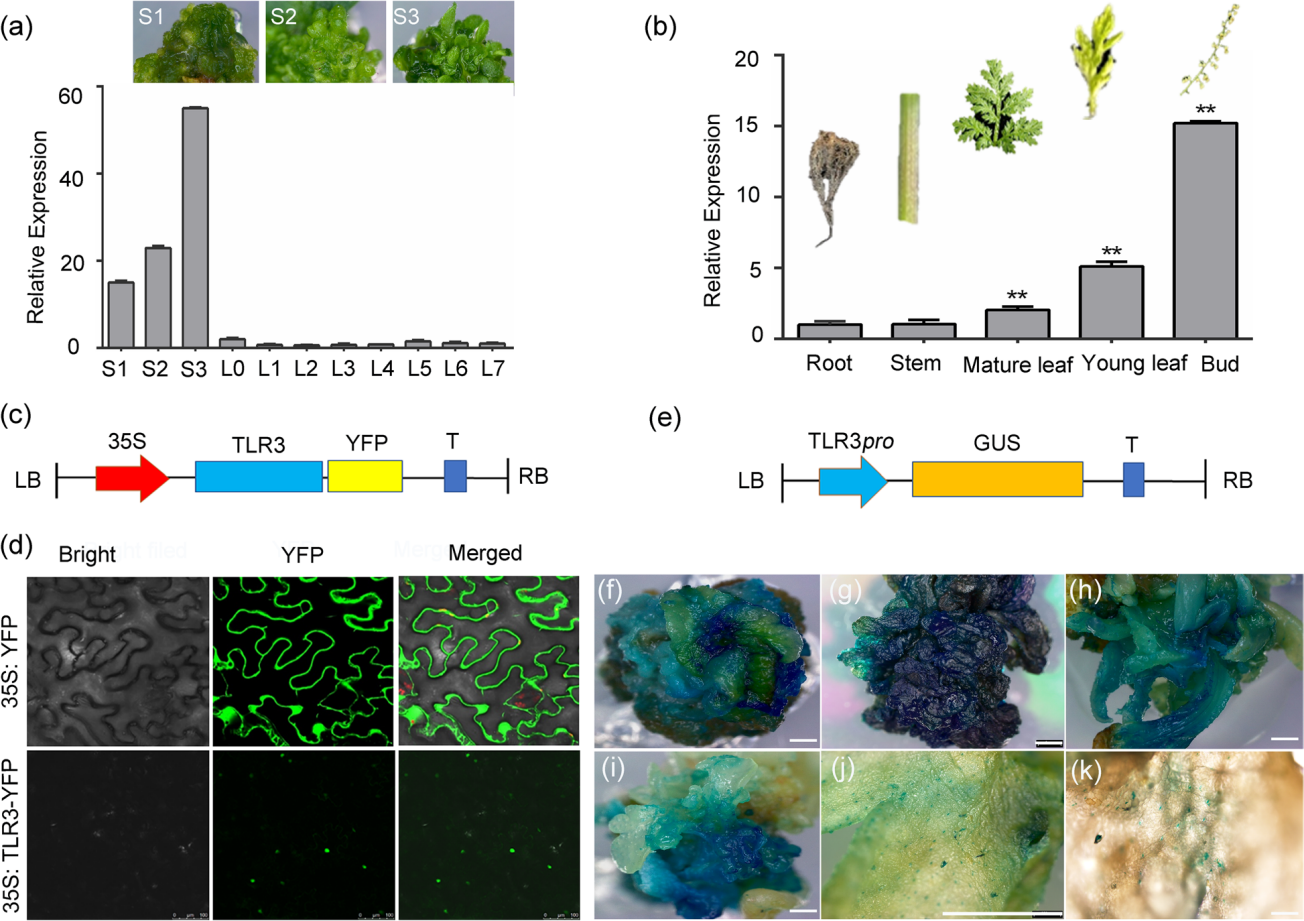
*TLR3* expression level and TLR3 subcellular localization in *A*. *annua*. **a** Relative *TLR3* expression level in callus (stages S1–S3) and leaves 0–7 (L1–L7). S1, callus primary stage; S2, callus middle stage; S3, shooting stage. **b** Relative *TLR3* expression level in roots, stems, old leaves, young leaves and flowers. **c** Schematic of the *TLR3-YFP* construct, driven by the 35S promoter. YFP, yellow fluorescent protein; LB, left border; RB, right border; T, termination sequence. **d** TLR3 is localized in the nucleus in *Nicotiana benthamiana* leaf epidermal cells. Free YFP (*35S:YFP*) was used as a control. **e** Schematic of the *TLR3pro:GUS* reporter construct. *GUS*, *ß-glucuronidase*. **f**–**k** GUS staining of *A. annua* callus and tissues from *TLR3pro:GUS* transgenic materials. Scale bars, 1 mm

To elucidate the functions of *TLR3*, we assessed its expression levels across various tissues and developmental stages. We gathered diverse tissues, including callus (derived from tissue culture) at different stages (S1, primary stage; S2, middle stage; S3, shooting stage), various leaves (leaf 0 to leaf 7) as well as roots, stems, and buds. Our analysis revealed a high expression of *TLR3*, particularly during the callus stage, with peak expression observed in S3 (Fig. 2a). Significantly, this heightened expression aligns with an increase in trichome number during this particular stage. *TLR3* exhibits elevated expression levels in young leaves and buds compared to mature leaves (Fig. 2b), supporting its putative role in trichome development. To further investigate its subcellular localization, we engineered a construct encoding TLR3 fused to the yellow fluorescent protein (YFP) and transiently expressed in *Nicotiana benthamiana* epidermal cells. Through confocal laser scanning microscopy, we detected YFP fluorescence in the nucleus, consistent with the anticipated behavior of a transcription factor (Fig. 2c, d). As a complementary strategy, we inserted the *TLR3* promoter upstream of the *ß-glucuronidase* (*GUS*) reporter gene and created transgenic *A. annua* plants through stable transformation. Strong GUS staining was evident at the S3 stage, while younger seedlings displayed a comparatively lower staining intensity (Fig. 2e–j). The control nontransgenic callus exhibited no staining (Fig. S1), corroborating the specificity of GUS staining associated with *TLR3* expression, as detected in various callus stages through RT-qPCR (Fig. 2a). In mature transgenic plants, GUS staining from the *TLR3pro:GUS* reporter was generally less pronounced than in seedlings and predominantly localized to the stoma between pairs of guard cells (Fig. 2k). This observation suggests that *TLR3* may have additional functions beyond its role in trichome development. In summary, our findings indicate that *TLR3* is predominantly expressed during the callus stage, coinciding with the observed increase in trichome number at this developmental stage.

### *A. annua* TLR3 negatively regulates trichome density and branching in *Arabidopsis*

**Fig. 3:**
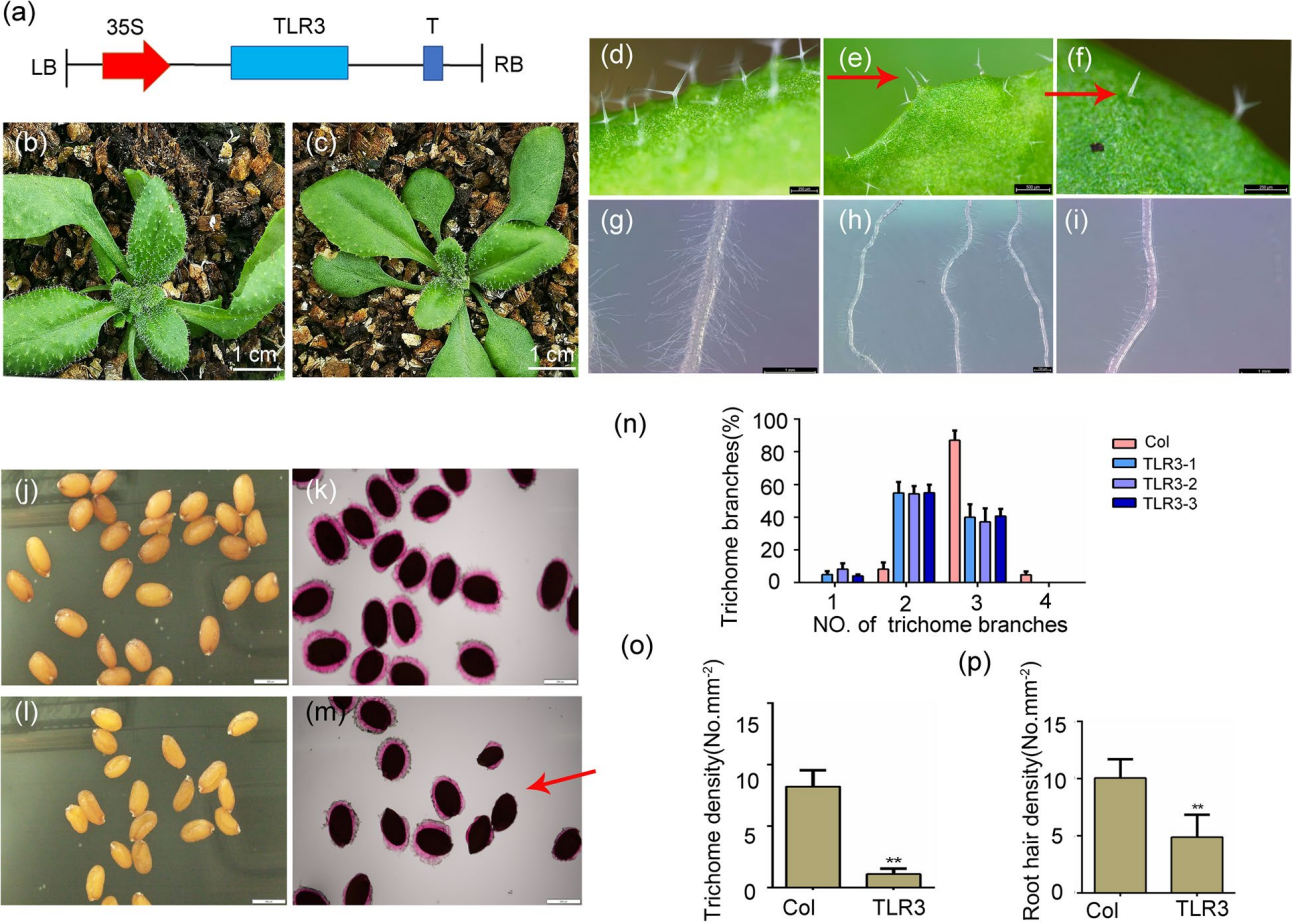
Heterologous expression of *TLR3* in *Arabidopsis thaliana* represses trichome development*.* **a** Schematic of the *35S:TLR3-PHB* construct. *PHB* is the vector used for gene overexpression in plants. **b** Representative nontransgenic Col-0 plant. **c** Representative *TLR3-*OE plant*.* **d**–**n** Transgenic plants show fewer trichomes, and trichomes have fewer branches (**e**, **f**, **n**). Red arrows point to a trichome with a single branch (**e**) and a trichome with two branches (**f**). As compared to Col-0 (**g**), the transgenic plants have a lower density of root hairs (**h**, **i**). Seeds of Col-0 and transgenic plants, without (**j**, **l**) and with (**k**, **m**) ruthenium red staining. The transgenic seeds are abnormal seeds, with a defect in mucilage (stained by ruthenium red; m). **n** Distribution of the number of trichome branches. **o** Distribution of the number of trichome density. **p** Distribution of the number of root hair density. Asterisks indicate significant differences relative to Col-0 (Student’s *t*-test; **, *P* < 0.01). Data are means ± SE

To confirm the involvement of *A. annua TLR3* in trichome development, we introduced the gene in *Arabidopsis*. Remarkably, we observed a suppression of trichome formation in *TLR3*-overexpressing (*TLR3-*OE) lines, as indicated by a reduced trichome density compared to the wild-type Col-0, particularly noticeable along the midvein region of leaves (Fig. 3a–c, n-p). Remarkably, *TLR3* exhibited a splice variant containing an intron, which we designated as *TLR3-intron*. (Fig. S2a, b). We introduced *TLR3-intron* (*TLR3i*) into *Arabidopsis* through heterologous expression and observed a more pronounced phenotype, characterized by a significantly lower trichome density in transgenic lines compared to *TLR3-*OE lines (Fig. S2b). *TLR3-*OE plants exhibited an altered trichome morphology, producing more trichomes with one or two branches, as opposed to the typical three branches observed in Col-0 (Fig. 3d–f, n-p). Additionally, four-branch trichomes, occasionally observed in Col-0, were absent in *TLR3-*OE lines. Moreover, *TLR3-*OE lines displayed a reduced density of root hairs (Fig. 3g–I, n-p). To gain insights into the molecular basis of these phenotypic changes, we examined the expression of known regulators of trichome and root hair development in 4-week-old *TLR3*-OE *Arabidopsis* plants: *CPC* and *TRY* (important negative regulators of trichome development), along with *WER* (*WEREWOLF*), *ZHD8* (*ZINC FINGER HOMEODOMAIN 8*) and *SCN1* (*SUPERCENTIPEDE 1*) (important negative regulators of root hair development). RT-qPCR analysis revealed upregulation of *CPC*, *WER*, *ZHD8*, *SCN1* and *TRY* expression in the *TLR3*-OE lines (Fig. S3), providing molecular evidence consistent with the observed trichome and root hair phenotypes in Arabidopsis *TLR3-*OE lines.

Examining the seeds of these transgenic lines, we observed that the overall morphology of the seed coat of *TLR3*-OE lines resembled that of Col-0. Typically, Col-0 seeds are enveloped by a thick mucilage layer that stains with ruthenium red. However, seeds from the *TLR3*-OE transgenic lines exhibited a thinner or discontinuous mucilage layer (Fig. 3j–m). Furthermore, *TLR3*-OE plants exhibited reduced secondary branches (secondary branches) and a smaller overall size compared to Col-0 (Figs. S4 and S5), with only approximately half the fresh weight of Col-0 plants (Fig. S6). Therefore, it can be concluded that *TLR3* acts as a negative regulator for trichome density and branching in *Arabidopsis*.

### TLR3 physically interacts with NFY1 and CycTL

**Fig. 4:**
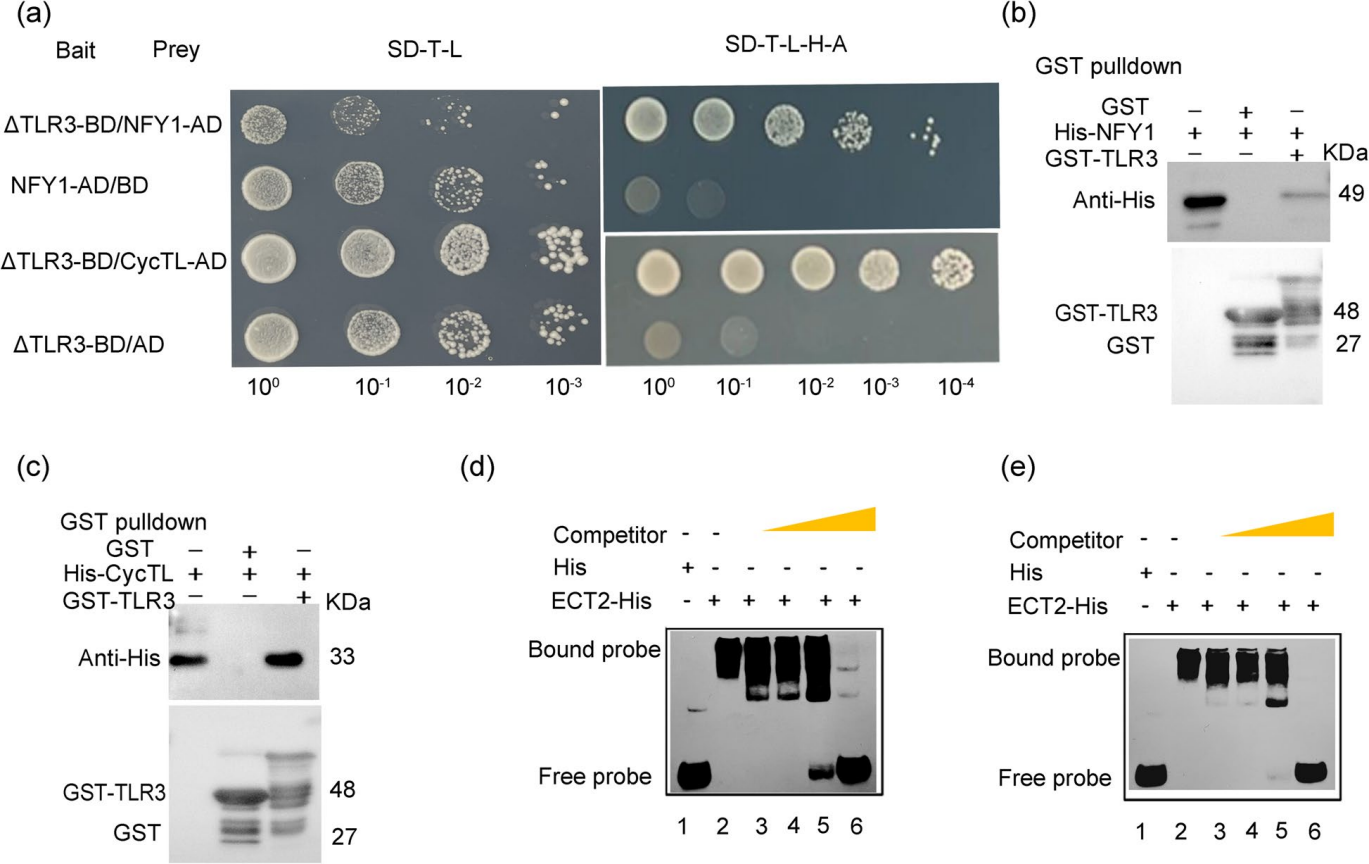
TLR3 can interact with NFY1 and CycTL in yeast two-hybrid and pull-down assays and can bind to the UGUAA, UGUAU and GGACU of *ECT2* mRNA. **a** Yeast two-hybrid (Y2H) assay indicating that truncated TLR3 (∆TLR3, amino acids [aa] 1–111) interacts with NFY1 and CycTL. The bait and prey plasmids were co-transformed into yeast and positive colonies were selected on synthetic defined (SD) medium lacking Trp, Leu, His and Ade. **b**–**d** Pull-down assay indicating that AaTLR3 interacts with NFY1 (**b**), CycTL (**c**). Recombinant GST-TLR3, His-NFY1 and His-CycTL were produced in *E*. *coli* Rosetta (DE3) and purified. After incubation in vitro, the protein mixture was separated by SDS-PAGE gel. Proteins were detected with anti-His and anti-GST antibodies. **d**, **e** ECT2-His interacts with the UGUAA, UGUAU and GGACU of *TLR3* mRNA on RNA-EMSA. Recombinant ECT2-His was produced in *E*. *coli* Rosetta (DE3) and purified. The concentration ratios of free probe and bound probe in the third to sixth lane were 1:1, 1:10, 1:100 and 1:1000, respectively

The aforementioned findings suggest that TLR3 may play crucial roles in regulating trichome development. The amino acid sequence encoded by TLR3 was submitted to the Pfam website (http://pfam.xfam.org/) for structure prediction. The gene comprises two conserved MYB domains within the range of 1–111 amino acids. Consequently, the gene was divided into three segments: full-length amino acids, 1–111 amino acids, and 112–206 amino acids, designated as TLR3-full, ΔTLR3, and ΔTLR3’. Primers were designed for cloning, and the BD vector pGBKT7 was constructed through homologous recombination, followed by transformation into Escherichia coli and sequencing. The correct plasmid was extracted and subjected to self-activation detection. Based on the results from the four missing plates (-T-L–H-A) (S7), BD-TLR3 full length (BD-TLR3 full) exhibited a self-activation phenomenon that was challenging to inhibit. BD-ΔTLR3 showed no self-activation phenomenon, representing the MYB domain fragments. The BD-ΔTLR3’ sequence displayed self-activation, suggesting that this segment may be a self-activated domain. Therefore, We deleted the transcriptional activation domain of TLR3 and generated truncated version of TLR3, ΔTLR3 (amino acids [aa] 1–111), to screen a cDNA library generated from total RNA extracted from *A. annua* leaves by yeast two-hybrid (Y2H) to identify interacting partners. Numerous proteins interacted with ΔTLR3, including a NUCLEAR FACTOR Y (NFY) protein, designated as NFY1. We verified the interaction between ΔTLR3 and NFY1 through direct Y2H assay (Fig. 4a) and in a glutathione S-transferase (GST) pull-down assay involving recombinant GST-TLR3 and His-tagged NFY1 (Fig. 4b). CycTL is known to be involved in cuticular wax loading and negatively regulates trichome development in both *A. annua* and *Arabidopsis* (Dong et al. 2021). Therefore, we also investigated whether ΔTLR3 might interact with CycTL (Fig. 4a, c), Indeed, Y2H and pull-down assays supported the notion that TLR3 interacts with CycTL.

### TLR3 regulates trichome branching

Previous studies have demonstrated that trichome branching in *Arabidopsis* can be regulated by the protein ECT2, a conserved protein capable of binding to 6-methyladenosine (m6A)-containing RNAs in vivo through its YTH (YT521-B homology) domain (Scutenaire et al. 2018; Wei et al. 2018). In our investigation, we reconstructed a phylogenetic tree of ECT2 from *Arabidopsis* and other plant species, identifying a putative ECT2 ortholog in *A. annua* (Fig. S8). AtECT2 exhibits a specific binding affinity for the m6A-modified sequence URUAY in *Arabidopsis* mRNAs. Remarkably, the *TLR3* mRNA also contains this motif, prompting us to hypothesize that AaECT2 may likewise bind to the *TLR3* mRNA. To investigate this hypothesis, we conducted an RNA electrophoretic mobility shift assay (EMSA) using RNA probes labeled with digoxigenin. Our findings indicate that ECT2 binds to the sequences UGUAA, UGUAU and GGACU present in the *TLR3* mRNA (Fig. 4d, e). Consequently, we infer that trichome development is contingent upon the functional role of ECT2.

### *TLR3* negatively modulates trichome development and artemisinin yield in *A. annua*

**Fig. 5:**
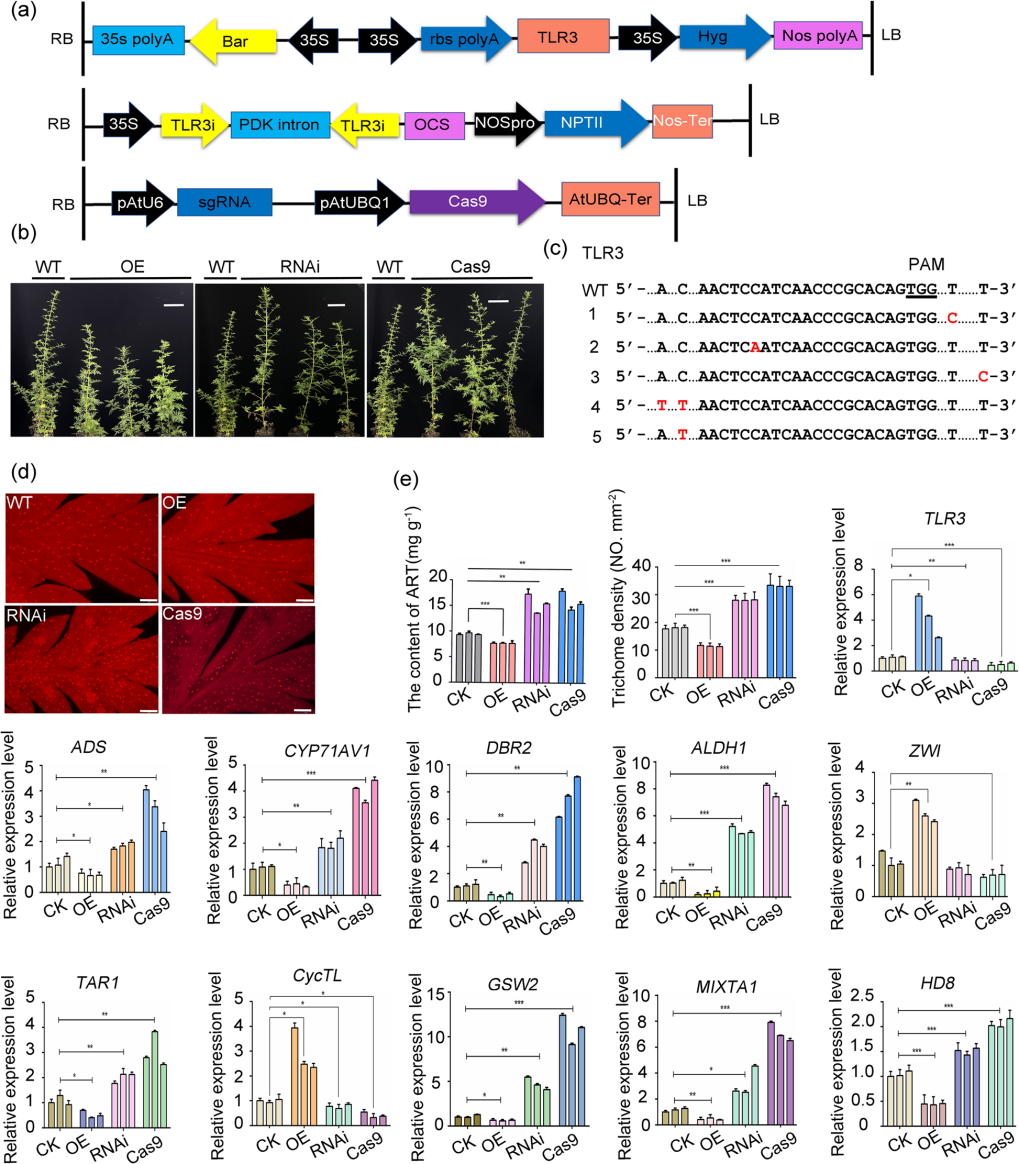
Phenotypes and relative expression of genes in transgenic *A*. *annua* plants. **a** Schematics of the vectors PHB-TLR3, RNAi-*TLR3* and Cas9-*TLR3*. **b** Comparison of plant height in WT and *TLR3* transgenic plants. Scale bars, 10 cm. **c** Identification of *TLR3* mutations in *TLR3*-Cas9 plants. The bases in red are insertions generated by genome editing. **d** Trichome density on the leaves of WT, *TLR3*-OE, *TLR3*-RNAi and *TLR3*-Cas9 plants as visualized by fluorescence microscopy. Scale bars, 100 µm. **e** Content of ART (artemisinin), trichome density and relative expression levels of the indicated genes in transgenic *TLR3 A*. *annua* plants

As demonstrated earlier, we observed that *TLR3* exerts a negative regulatory effect on trichome development when introduced in *Arabidopsis* and interacts with cuticle-related proteins from *A. annua* in yeast. To further explore the potential involvement of *TLR3* in trichome and cuticle development in *A. annua*, we generated transgenic *A. annua* plants overexpressing *TLR3* (*TLR3-*OE), plants with *TLR3* mRNA knockdown (*TLR3*-RNAi), and plants expressing a mutant TLR3 created through clustered regularly interspaced short palindromic repeat (CRISPR)/CRISPR-associated nuclease 9 (Cas9)-mediated editing using *Agrobacterium tumefaciens*-mediated transformation (Cas9-*TLR3*; Fig. 5a, b). Sanger sequencing revealed the successful editing of five *A. annua* plants (Fig. 5c).

To evaluate the impact of *TLR3* on trichome development in *A. annua*, we examined the transcript levels of *TLR3*, trichome development genes and artemisinin biosynthesis genes in these three groups of plants. In the *A. annua TLR3*-OE lines, *TLR3* expression increased approximately 3- to 6-fold, while the overall expression of artemisinin biosynthesis genes was reduced (Fig. 5e). Moreover, artemisinin levels were approximately 16–21% lower than in control lines, suggesting that *TLR3* acts as a negative regulator in artemisinin biosynthesis. Conversely, the *A. annua TLR3*-RNAi and gene-edited lines exhibited an expression pattern opposite to that of the *TLR3-*OE lines. Trichome density analysis across all lines revealed a 38%–42% reduction in density in *TLR3-*OE lines compared to control lines (Fig. 5d, e). However, trichome density increased by 45%–69% in the *TLR3*-RNAi lines and by 75%–100% in the gene-edited lines. Artemisinin content showed a 60%–80% increase in *TLR3*-RNAi lines and a 60%–90% increase in gene-edited lines (Fig. 5e). These findings strongly support the conclusion that TLR3 plays a role in both trichome development and artemisinin accumulation.

### Overexpression of TLR3 leads to abnormal trichomes in *A. annua*

**Fig. 6:**
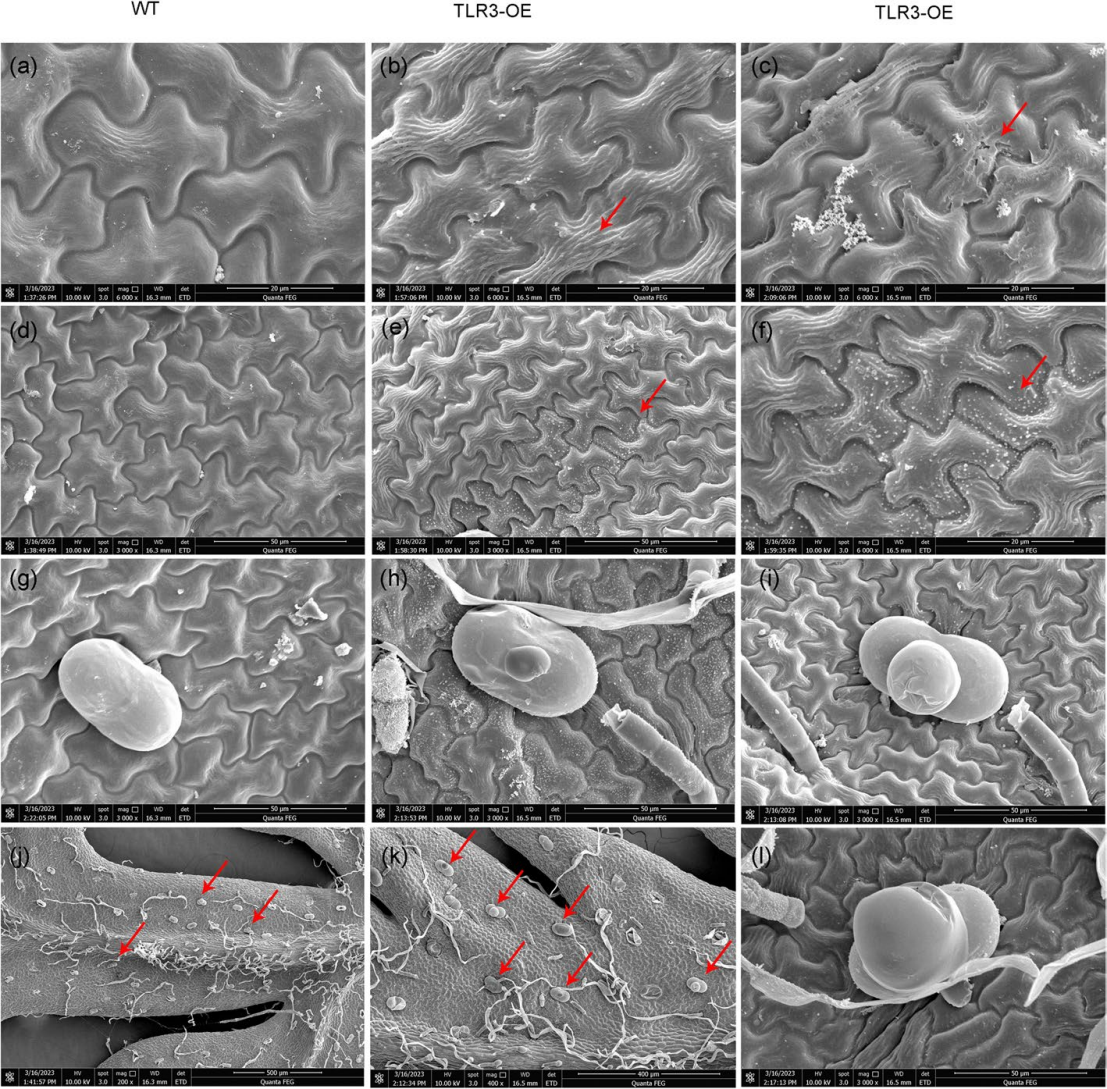
Scanning electron microscopy (SEM) analysis of the cuticle and trichomes on the surface of *A. annua* leaves. **a** Smooth surface of WT *A. annua* leaves. **b** Coarse and fibrous surface of *A. annua TLR3*-OE leaves. **c** A *TLR3*-OE leaf with detached cuticular wax visible on its surface. **d** Smooth surface of WT *A. annua* leaves without white wax crystals. **e**, **f** *TLR3*-OE leaves show uneven distribution of white wax crystals. **g** A normal glandular trichome on a WT *A. annua* leaf. (**h**, **i**, **l**) Abnormal glandular trichome on a leaf of an *A. annua TLR3*-OE plant. **j** Few abnormal glandular trichomes (red arrows) are visible on WT *A. annua* leaves. **k** Many abnormal glandular trichomes (red arrows) are visible on the leaves of *A. annua TLR3*-OE plants

Considering *TLR3*’s role as a negative modulator in trichome branching development in *Arabidopsis*, we hypothesized that TLR3 might similarly influence trichome morphogenesis in *A. annua*. To explore this, we examined whether *TLR3* overexpression would suppress trichome branching by examining non-glandular trichomes using fluorescence microscopy, as these structures exhibit strong autofluorescent. Surprisingly, *A. annua TLR3*-OE plants did not display abnormalities in either non-glandular or glandular trichomes (Fig. S9). To delve deeper into the trichome morphogenesis of *TLR3*-OE plants, in more detail, we utilized scanning electron microscopy (SEM). We observed numerous calabash-like trichomes on the leaf surface of *A. annua TLR3-*OE lines (Fig. 6j, l), in contrast to the sparse occurrence of club trichomes in the wild-type control (Fig. 6i, k). Furthermore, non-glandular trichomes appeared smaller in the *TLR3-*OE lines (Fig. S10a), but larger in the *TLR3-*OE lines compared to the wild type (Fig. S10b). We propose that the inhibition of trichome development does not impede the underlying metabolic flux, resulting in larger trichomes capable of depositing secondary metabolites. In summary, these findings demonstrate that TLR3 has the ability to influence the visual characteristics of trichomes in *A. annua*.

### Overexpression of TLR3 alters cuticle biosynthesis in *A. annua*

SEM analysis of the leaf surface unveiled a fibrous texture with a rugged appearance in the epicuticular layer of *A. annua TLR3-*OE lines (Fig. 6a–d). Furthermore, we noted a reduction in the size of epidermal cells in *A. annua TLR3*-OE lines compared to wild type (Fig. 6a, b, Fig. S11). The *TLR3-*OE leaves exhibited numerous non-glandular trichomes on their surface, a feature absent in wild-type leaves (Fig. 6e, f). Additionally, wax crystals on the epicuticular layer displayed a uniform distribution in the *TLR3-*OE lines, accompanied by clear evidence of increased fragility when compared to the wild type (Fig. 6g, h).

**Fig. 7:**
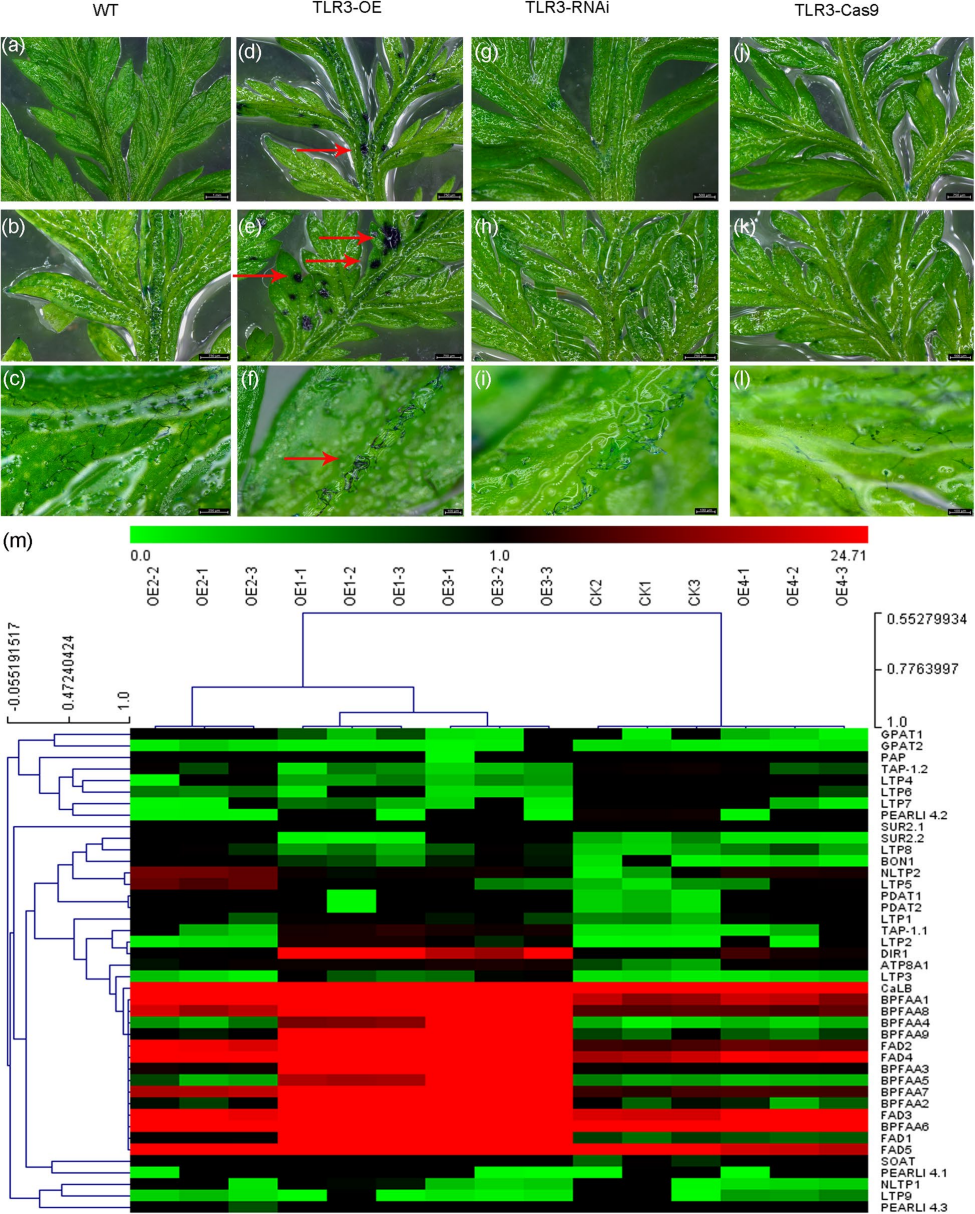
*TLR3* is involved in the biosynthesis of cuticle in *A. annua.* **a**–**l** Young leaves of WT (**a**), *TLR3*-OE (**d**), *TLR3*-RNAi (**g**) and *TLR3*-Cas9 (**j**) plants treated with toluidine blue (TB); mature leaves of WT (**b**), *TLR3*-OE (**e**), *TLR3*-RNAi (**h**) and *TLR3*-Cas9 (**k**) plants treated with TB; and leaf veins of WT (**c**), *TLR3*-OE (**f**), *TLR3*-RNAi (**i**) and *TLR3*-Cas9 (l) plants were treated with TB. Red arrows point to sites that took up toluidine blue. **m** Hierarchical clustering analysis of genes involved in biosynthesis of lipids in WT *A. annua* and *TLR3*-OE leaves. The color scale at the top represents the log-transformed reads per kilobase per million mapped reads

An evident modification in the protective cuticular layer of leaves was observed through toluidine blue (TB) staining, a method known to selectively highlight leaves with a compromised cuticle (Tanaka et al. 2004). Staining was notably present in the leaves of *A. annua TLR3*-OE lines (Fig. 7a–l), serving as a clear indicator of cuticular defects.

These findings suggest a notable alteration in the cuticle composition of *A. annua TLR3-*OE line leaves. To elucidate the underlying factors contributing of these differences, we conducted an RNA-seq analysis of *A. annua* wild-type and *TLR3*-OE lines. Upon mapping sequencing reads to the *A. annua* reference genome (Table S3), we identified 3,047 genes exhibiting differential expression between *TLR3*-OE and control plants. Among the top 500 most significant genes distinguishing WT and the OE lines, seven genes encoding proteins involved in lipid biosynthesis stood out: sphingolipid C4 hydroxylase (SUR2) (2 genes), phospholipase-like protein (PEARLI 4) (3 genes), lipid transfer protein (LTP) (1 gene), and bifunctional inhibitor/plant lipid transfer protein/seed storage helical domain-containing protein (DIR1) (1 gene). Notably, no genes associated with wax biosynthesis were among the differentially expressed genes (Table S4), prompting us to posit that TLR3 primarily influences lipid biosynthesis in *A. annua*. The expression patterns of 42 genes implicated in lipid biosynthesis were visually represented in *A. annua* wild-type and *TLR3*-OE lines (Fig. 7m).

As the plant cuticle primarily consists of long-chain fatty acids, we conducted quantification of these fatty acids in the plant leaves using LC–MS/MS (Hodek et al. 2022). In comparison to the wild type, *A. annua TLR3*-OE lines exhibited significantly elevated levels of C20 lyso-diacylglyceryltrimethylhomoserine (LDGTS), C24-32 free fatty acids (FFA), C32 disaccharide diglycerides (DGDG), C32-44 diglyceride (DG), C34 acyl diacylglycerol glucuronic acid (ADGGA), C34 monosaccharide diglycerides (MGDG) and phosphatidic acid C32-36 (PA) (*P* < 0.05) (Fig. S12).

In the *TLR3-*OE lines, several lipids were found to be down-regulated relative to wild type. These included diacylglyceryltrimethylhoserine (DGTS) at C39, ceramide (Cer) at C32-45, phosphatidylcholine (PC) at C32/C34, phosphatidylethanolamine (PE) at C32-38, phosphatidylinositol (PI) at C36 and phosphatidylserine (PS) at C32-37 (*P* < 0.05).

Furthermore, the levels of triglyceride (TG) and phosphatidylglycerol (PG) with varying carbon chain lengths at C35-60 and C31-36 demonstrated significant increases or decreases when compared to the WT (*P* < 0.05). Collectively, these findings suggest that *TLR3* has the capacity to modulate the expression of genes associated with lipid biosynthesis, consequently influencing lipid composition and inducing alterations in the cuticle in *A. annua*.

## Discussion

### TLR3 negatively regulates biomass by interacting with NFY1

Previous studies have suggested that MYB TFs can affect plant biomass (Fávero et al. 2018; Zheng et al. 2023). In this investigation, we demonstrate that *TLR3* exerts a negative regulatory influence on biomass in *Arabidopsis* when expressed heterologously (Fig. S6), aligning with prior findings. Root hairs play a crucial role in nitrogen uptake, promoting plant growth and development, and consequently, biomass accumulation (De Pessemier et al. 2022). *Arabidopsis TLR3*-OE lines exhibited a reduction in the number of root hairs compared to the wild-type Col-0, indicating a diminished uptake of essential nutrients from the medium or soil in these lines (Fig. 3g–i, n-p). Through yeast two-hybrid (Y2H) and pull-down assays, we established the interaction between TLR3 and NFY1 (Fig. 4a, b). NFY1 belongs to the NF-Y family of CCAAT-box binding TFs (Hackenberg et al. 2012) and has been shown to regulate pyruvate yield, influencing metabolic flux to the tricarboxylic acid (TCA) cycle (Ke et al. 2022). Certainly, our RNA-seq analysis comparing CK and *TLR3*-OE lines in *A.annua* revealed the downregulation of key genes associated with the TCA cycle. Notably, *Pyruvate kinase 2* (*PK2*, chr3g00571631), *Succinate dehydrogenase subunit 4* (*SDH*), *Citrate synthase* (*CS*) and *Phosphoenolpyruvate carboxylase* (*PEPC*), suggesting that the flux to the TCA is downregulated (Fig. S12). This observation leads us to the conclusion that TLR3 likely modulates the TCA cycle, thereby influencing plant biomass.

### TLR3 negatively regulates the density and branching of trichomes

MYB TFs serve as crucial constituents of MBW (MYB, bHLH, WD40) complexes, playing a pivotal role in trichome development in *Arabidopsis* (Ishida et al. 2008) and other plant species, including tomato, *A. annua* and cotton (*Gossypium hirsutum*) (Shi et al. 2018; Wang et al. 2021; Yuan et al. 2021). It is noteworthy that several MYB TFs exhibit negative regulatory roles in trichome development, exemplified by TRY, CPC and ETC1 (Schellmann et al. 2002; Kirik et al. 2004a, b). In our phylogenetic analysis, *A. annua TLR3* clustered closely with *Arabidopsis* MYB5 (Fig. 1f), a potential involvement in trichome development. Indeed, the heterologous expression of *TLR3* in *Arabidopsis* resulted in a negative regulation of trichome density, indicating a functional similarity between *TLR3* and *AtMYB5* (Fig. S2). Furthermore, our findings demonstrate an interaction between TLR3 and CycTL, a protein known to be associated with trichome development in *A. annua* (Dong et al. 2021). These two proteins interact to influence the accumulation of artemisinin by affecting the wax load in the leaf epidermis, altering trichome initiation, and downregulating trichome density. Upon overexpressed *TLR3* in *A. annua*, the expression of several genes encoding positive regulators of trichome development decreased, while the expression of genes encoding negative regulators increased (Fig. 5e). Furthermore, trichome density decreased in *A. annua TLR3*-OE lines but increased in *TLR3*-RNAi lines (Fig. 5e). In *Arabidopsis TLR3*-OE lines, there was an observed increase in trichomes with one or two branches, deviating from the typical three branches in Col-0 (Fig. 3d–f). Collectively, these findings lead to the conclusion that *TLR3* plays a negative regulatory role in modulating both the branching and density of trichomes.

### TLR3 modulates cuticle biosynthesis in *A. annua*

The trichome surface is enveloped by a layer of cuticle (Duke and Paul 1993; Lv et al. 2021). Altering cuticle abundance or composition of the cuticle may impact trichome density in *A. annua* (Tan et al. 2015), highlighting the crucial role of cuticle biosynthesis in trichome development. In *Arabidopsis*, *MYB16* and *MYB106* have been identified as contributors to cuticle component synthesis. This was evident when each gene was fused to a sequence encoding a repressor SRDX motif, resulting in observable defects in cuticle development, as visualized by toluidine blue (TB) stain uptake (Oshima et al. 2013). TB staining revealed a defective cuticle in *A. annua TLR3*-OE lines compared to the wild type (Fig. 7a–l). Consistent with this observation, RNA-seq analysis of *A. annua TLR3*-OE lines identified seven genes associated with lipid biosynthesis (not wax biosynthesis) among the top 500 most significant genes (Table S3), suggesting that TLR3 primarily regulates lipid biosynthesis. Since cuticle originates from very-long-chain fatty acid (VLCFA) precursors (Seo et al. 2011; Gonzales-Vigil et al. 2021), we quantified the abundance of these compounds in the leaves of wild-type and *A. annua TLR3*-OE plants. Lower levels of fatty acids, including DGTS (39 carbons), Cer (32–45 carbons), PC (32–34 carbons), PE (32–38 carbons), PI (36 carbons) and PS (32–37 carbons), were detected in *A. annua TLR3*-OE plants compared to the wild type. This indicates that TLR3 is indeed involved in the biosynthesis of fatty acids in *A. annua*.

*A. annua TLR3*-OE leaves exhibited an uneven distribution of cuticular white wax crystals on their surface compared to wild type (Fig. 6c, d), indicating the involvement of TLR3 in cuticular wax formation. Additionally, we found that TLR3 interacts with CycTL, a known regulator of cuticle biosynthesis (Dong et al. 2021) (Fig. 4a, c). These findings unequivocally establish that TLR3 modulates wax biosynthesis in *A. annua*. Previous studies have suggested that mutations in genes associated with cuticle biosynthesis can impact the development or growth of plant organs (Nobusawa et al. 2013). In the case of *A. annua TLR3*-OE plants the presence of numerous abnormal trichomes (Fig. 6k, l) may be attributed to the altered cuticle in these plants.

### Mode of trichome and cuticle regulation by TLR3

We have established that the putative TF TLR3 interacts with CycTL and NFY1 to regulate cuticle biosynthesis, trichome morphogenesis and biomass in *A. annua*. Previously, we demonstrated that CycTL interacts with the TF TAR1, disrupting cuticle arrangement in both *Arabidopsis* and *A. annua* (Tan et al. 2015; Dong et al. 2021). The coverage of the leaf surface by cuticle can impact trichome development in various plants including *Arabidopsis*, *A. annua* and tomato (Hegebarth et al. 2016; Dong et al. 2021). However, it remains unknown whether TLR3 directly or indirectly activates the expression of genes involved in cuticle biosynthesis. Identifying direct target genes of *TLR3* and investigating their roles in cuticle biosynthesis in *A. annua* will be an intriguing avenue for future research. Genes such as *SlCER6*, *SlKCR1*, *SlPAS2* and *SlLTP*, known for their involvement in wax biosynthesis and trichome development, emerge as promising candidates (Xiong et al. 2020).

TLR3 interacted with NFY1, exerting a negative regulatory impact on plant biomass in *Arabidopsis* (Fig. S6) and influencing cell size in *A. annua* (Fig. S11). The NF-Y family modulates biomass by impacting the TCA cycle (Ke et al. 2022). Notably, the majority of genes associated with the TCA cycle were found to be downregulated in the *A. annua TLR3*-OE lines (Fig. S13). Several potential candidate genes linked to the TCA cycle, including *PK2*, *SDH*, *CS* and *PEPC*, have the potential to influence biomass and thus represent plausible target genes for NFY1.

In the context of trichome morphology, numerous proteins play a role in trichome branching, including TCP, TRY, CPL3 (CAPRICE-LIKE MYB3), GL3, CYCD3;1 (CYCLIN D3;1), and CYCB1;2 (Schnittger et al. 2002a, b; Schnittger et al. 2002a, b; Esch et al. 2003; Camoirano et al. 2020). However, pinpointing the specific candidate gene responsible for trichome branching will necessitate additional investigation.

**Fig. 8:**
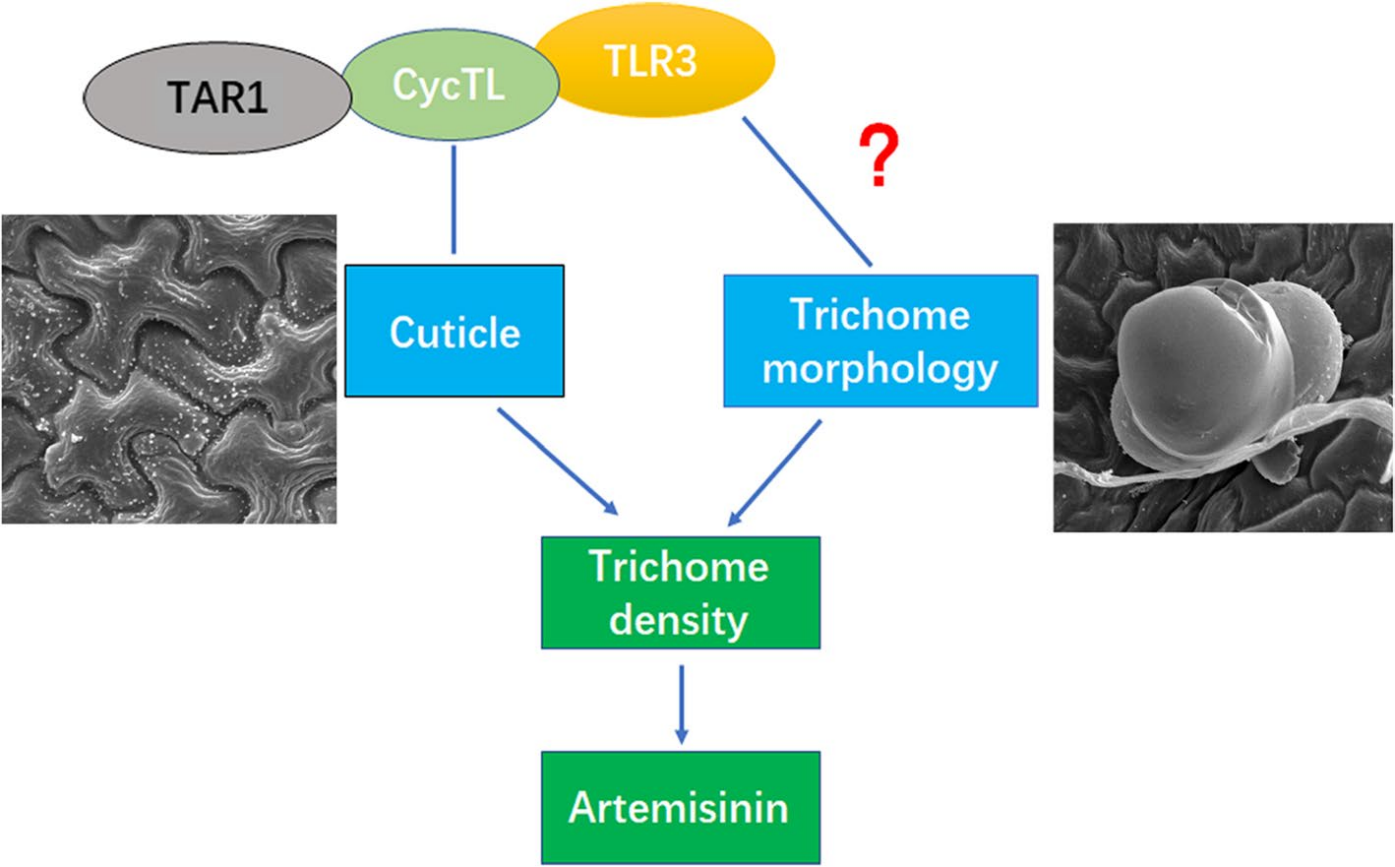
Model for TLR3 regulation of cuticle and trichome morphology. TLR3 interacts with CycTL, which interacts with TAR1. The three proteins may regulate the distribution of cuticle on *A. annua* leaves. TLR3 also affects trichome morphology, resulting, for instance, in club trichomes, calabash trichomes. During trichome development, genes influence cuticle biosynthesis and trichome morphology, altering the initial and phenotype of trichome in *A. annua*

Future research will concentrate on identifying the downstream target genes of CycTL and NFY1 to unravel the regulatory network governing cuticle biosynthesis, biomass, and trichome morphology. Furthermore, investigation into the upstream factor regulating *TLR3* expression is warranted. A working model illustrating this complex is depicted in Fig. 8.

## Conclusion

Our findings suggest that *TLR3* serves as an negative regulator for trichome initiation in *A.annua*. Down-regulation of *TLR3* may potentially enhance trichome density and elevate artemisinin content. *TLR3* exerts its influence on trichome morphology by modulating cuticle development. Consequently, *TLR3* emerges as a crucial molecular marker for trichome density and artemisinin yield.

## Materials and methods

### Plant material

The seeds of *Artemisia annua* L. ‘huhao 1’ were surface sterilized in 10% (v/v) sodium hypochlorite (NaClO) for 5 min, followed by rinsing with sterile water three times. These seeds were then sown onto Murashige and Skoog (MS) (Murashige and Skoog 1962) solid culture medium, which included 20% (w/v) sucrose, 7% (w/v) agar, and 0.043% (w/v) MS basal salts at pH 5.8. The seedlings were cultivated at 24 °C under a 16 h: 8 h light: dark photoperiod with a light intensity of 7500 lx. Surface-sterilized *Arabidopsis* seeds (Columbia-0) were also grown on solid MS medium containing 20% (w/v) sucrose.

Seeds of *Nicotiana benthamiana* were planted in a soil mixture (vermiculite: perlite: peat moss, 7: 0.5: 3) (v/v) and plants were cultivated at 24 °C under a 16 h: 8 h light: dark photoperiod with a light intensity of 7500 lx.

### RNA isolation and RT-qPCR assay

Total RNA was isolated from different *A. annua* tissues using an RNAprep Pure Plant Kit (Tiangen Biotech) following the manufacturer’s instructions. The extracted total RNA was reverse transcribed into first-strand cDNA using a reverse transcription kit (cDNA synthesis kit, Code No. 6210A, TaKaRa Biotech) according to the manufacturer’s protocol. For the qPCR assay, a SYBR Green mix (Kapa Biosystems) was utilized, and the aforementioned first-strand cDNA served as template for measuring expression levels. *β-actin* was employed as an internal control, consistent with previous studies (Lv et al. 2016, 2019). The primer sequences used for RT-qPCR are detailed in Table S1.

### Yeast two-hybrid library screening

Total RNA was extracted from *A. annua* leaves and subsequently utilized to construct a yeast two-hybrid library in accordance with the manufacturer’s instructions. To investigate whether TLR3 could interact with other proteins, TLR3 was subjected to truncation, resulting in ΔTLR3 (which lacks the self-activation domain:112–206 amino acids). The encoding sequence of ΔTLR3 was cloned into the pGBKT7 plasmid (ΔTLR3-BD) and was subsequently transformed into the Y2H Gold yeast strain (serving as the bait). Both the bait and prey strains were introduced into the same yeast. Approximately 8.8 × 10^6^ yeast colonies were obtained and utilized to screen for positive clones on a selection medium (synthetic defined [SD]/–Trp/–Leu/–His/–Ade/X-α-Gal). The sequences of all identified positive clones were determined using Sanger sequencing.

### Yeast two-hybrid (Y2H)

For the Y2H assays, a GAL4-based two-hybrid system (Clontech) was utilized. The complete coding sequence of *TLR3* was cloned in-frame with the sequence encoding the activation domain of yeast GAL4 in the pGADT7 plasmid, generating a prey construct. The coding sequences of *Cycb3*, *NFY1* was subcloned into the pGADT7 plasmid to generate prey constructs. The primers used for cloning are detailed in Table S1. Both prey and bait vectors were co-transformed into yeast strain AH109 using the lithium acetate method (Gietz and Schiestl 2007). Transformants were cultured on SD medium lacking Trp or Leu (SD/–T/–L) for 4 d. Positive transformants were resuspended in sterile water and spotted onto SD/–Trp/–Leu or SD/–Ade/–Leu/–Trp/–His plates with different concentration: 10^0^, 10^–1^, 10^–2^, and 10^–3^. At least five individual colonies were analyzed for each bait/prey combination and cultured at 28 °C for 5 days.

### Pull-down assay

The coding sequence of *TLR3* was cloned into the pGEX4T-1 vector to facilitate the production of recombinant GST-tagged protein. Similarly, the coding sequences of *Cycb3*, *NFY1* and 7721 were subcloned into the pET32a vector to generate recombinant His-tagged proteins. All resulting clones were transformed into *Escherichia coli* strain BL21 and the production of the recombinant protein was induced at 18 °C for 12 h by adding 0.5 mM IPTG. The GST-tagged protein and His-tagged proteins were subsequently purified using Ni–NTA agarose beads (QIAGEN) and glutathione Sepharose beads (Amersham Biosciences), respectively. The GST-tagged proteins and His-tagged proteins were incubated at 4 °C for 3 h in binding buffer (300 mM NaCl, 20 mM Tris–HCl, 1% [v/v] Triton X-100, Cocktail [Roche, Code No. 4906845001]). The bound proteins were separated by SDS-PAGE, transferred to a PVDF membrane (Millipore Code No. IPFL00010), and detected by immunoblot analysis with anti-His (Abmart,1:5,000) and anti-GST (Abcam,1:5,000) antibodies.

### β-glucuronidase (GUS) assay

GUS activity assays were conducted following the protocol outlined in a previous study (Jefferson et al. 1987). The GUS staining buffer was composed of 50 mg X-Gluc (Merck, Code No.70036-M), 0.05 M Na_2_HPO_4_, 0.1% (v/v) Triton X-100, 10 mM EDTA. The tissues of *A. annua* were immersed in the GUS buffer and incubated at 37℃ in the dark for 12–24 h. To eliminate chlorophyll, the tissues were subjected to incubation in 70% (v/v) ethanol, with three consecutive replacements of fresh 70% ethanol. The treated tissues were examined using a Leica microsystems microscope (Switzerland, Model DVM6).

### RNA-EMSA

The His-tagged ECT2 protein was expressed in E. coli Rosetta (DE3) and subsequently purified using Ni–NTA agarose beads (QIAGEN). Biotin-labeled RNA transcripts were generated using the LightShift chemiluminescent RNA EMSA kit (Thermo, Waltham, MA, USA). The binding reactions were allowed to incubate for 30 min at room temperature. Following completion, 5μL of protein loading buffer was added. Subsequently, six samples of distinct labeled probes were loaded onto a 6% PAGE gel. The gel was run at a constant voltage of 100V until the bromophenol blue dye migrated to approximately three-quarters of the gel length. The gel assembly included a sequence of positive electrode—sponge—filter paper—nylon membrane—protein gel—filter paper—sponge—negative electrode. The transfer process was executed under conditions of 400 mA for 30 min, with an ice pack in the transfer liquid and the transfer instrument placed in an ice tank to prevent temperature elevation. Following the transfer, cross-linking with purple foreign link at a dosage of 120mJ/cm2 was immediately performed for one minute. The film was gently sealed with 20mL sealing solution at room temperature for 15 min. Subsequently, the sealer solution was replaced with a mixture of bond/sealer (20mL) for an additional 15-min sealing. To ensure cleanliness, four washes using a cleaning solution (1 ×) were conducted for five minutes each time on the film. This was followed by rinsing with substrate working buffer to ensure complete coverage over the entire film surface, followed by a five-minute incubation. Finally, color development was initiated using the developing liquid, and photographs were taken for record-keeping.

### Plant transformation

Transgenic *A. annua* plants were generated through Agrobacterium (*Agrobacterium tumefaciens*)-mediated transformation (Lv et al. 2022). Two-week-old *A. annua* seedlings served as explants for the procedure. Leaves of *A. annua* were excised and exposed to an Agrobacterium cell suspension in MS liquid medium (OD = 0.6) for 10 min, followed by co-cultured in the dark at 28 °C for 3 days. After this initial phase, all co-cultured leaves were transferred to shoot regeneration medium (MS medium, 0.5 mg L^–1^ 6-benzylaminopurine [6-BA], 0.05 mg L^–1^ naphthaleneacetic acid [NAA], 250 mg L^–1^ carbenicillin) at 25 °C for 3 weeks. The regenerated shoots were subsequently transferred to rooting medium (half-strength MS medium containing 50 μg ml^−1^ hygromycin B and 200 μg ml^–1^ temetine) for 1 month. Finally, the plants were planted into soil (vermiculite: perlite: peat moss, 7:0.5:3) (v/v) and grown at 24 °C under a 16 h: 8 h light: dark photoperiod with a light intensity of 7500 lx. DNA was extracted from young leaves of *A annua*, and the positive plants were identified by PCR using gene primers and carrier primers.

### Quantification of artemisinin using LC–MS/MS

*A. annua* samples were subjected to a drying process at 50 °C for three days, followed by grinding into powder. Subsequently, 0.1 g of each sample was accurately weighed, and extraction was performed using 2 mL of methanol. The supernatants were then analyzed for the presence of arteminisin (ART), artemisinic acid (AA) and dihydroartemisinic acid (DHAA) using liquid chromatography-tandem mass spectrometry (LC–MS/MS) (Tan et al. 2015).

### Lipid sample preparation and extraction

Lipids were extracted from *A. annua* leaves using a solvent mixture of methanol: methyl tert-butyl ether (MTBE) in a ratio of 1:3 (v/v). In brief, 20 mg of lyophilized powder and 1 mL of lipid extraction solvent (fortified with 0.1 μg/mL phosphatidylethanolamine [PE] 34:0 [17:0, 17:0] and phosphatidylcholine [PC] 34:0 [17:0, 17:0] as internal standards) were combined in 2-mL centrifuge tubes. One steel bead (internal diameter about 4 mm) was added, and the sample was vortexed for 30 min. Following this, 300 μL ultra-pure water was introduced, and the mixture was vortexed for an additional 1 min. Subsequently, a 10-min incubation without shaking at 4℃ took place. After centrifugation for 3 min at 12,000 rpm at 4℃, 400 μL of the supernatant was transferred to a 1.5-mL centrifuge tube and concentrated at 20℃ using SpeedVac until completely dry. Next, 200 μL of a lipid complex solution (1:1 (v/v) acetonitrile: isopropanol) was added to each tube, vortexed for 3 min, and then centrifugated for 3 min at 12,000 rpm at 4℃. Finally, 120 μL reconstituted sample was collected for LC–MS/MS analysis.

### Apparatus and LC–MS/MS conditions

LC–ESI–MS/MS analyses were conducted utilizing a UPLC system (ExionLC AD, https://sciex.com.cn/) in conjunction with a QTRAP® 6500 + System (https://sciex.com/). The separation of compounds was achieved through a gradient elution on a Thermo Accucore™ C30 column (2.6 μm, 2.1 mm × 100 mm i.d.) at 45℃. The mobile phase consisted of a binary gradient consisting of 0.1% (v/v) formic acid and 10 mM ammonium formate in H_2_O: acetonitrile (6:4, v/v) (A) with 0.1% (v/v) formic acid and 10 mM ammonium formate in isopropanol: acetonitrile (9:1, v/v) (B) at 0.35 mL/min. The gradient program followed these steps: starting at 20% B, it increased to 30% B from 0 to 2 min, then to 60% B from 2 to 4 min, further to 85% B from 4 to 9 min, to 90% B from 9 to 14 min, and to 95% B from 14 to 15.5 min. The gradient then held at 95% B from 15.5 to 17.3 min before concluding the analysis. Subsequently, a re-equilibration step of 2.7 min at 20% B was employed. A volume of 2 µl from each sample was injected for analysis.

Mass spectrometry detection was conducted on a QTRAP® 6500 + mass spectrometer (SCIEX, USA) employing both positive and negative electrospray ionization modes. The analysis conditions were set as follows: ion source with turbo spray; source temperature at 500ºC; ion spray voltage (IS) set to 5500 V (positive), –4500 V (negative); ion source gas 1 (GS1), gas 2 (GS2); curtain gas (CUR) set to 45, 55, and 35 psi, respectively. Instrument tuning and mass calibration were carried out utilizing 10 μM polypropylene glycol solutions in multiple reaction monitoring (MRM) mode.

## Statistical Information

The method of variance analysis of significant difference is T test, where *, *p* < 0.05; **, *p* < 0.01; ***, *p* < 0.001.

### Supplementary Information

**Additional file 1: Fig. S1. **Control calli cannot be stained.

**Additional file 2: Fig. S2.** Diagram of the *TLR3* locus and phenotype of *TLR3*-OE and *TLR3*-intron-OE lines. (a) The coding sequence of *TLR3 *is 621 bp in length; the length of the *TLR3*-intron is 834 bp. (b) Analysis of *TLR3* gene amplification by agarose gel electrophoresis. (c) TLR3-intron-OE lines have fewer trichomes than *TLR3*-OE plants.

**Additional file 3: Fig. S3.** Genes involved in trichome development and root hair development expression levels in *TLR3*-OE Arabidopsis lines. Data are means SD (*n* = 3). Asterisks indicate significant differences between *TLR3*-OE lines and Col-0 by Student’s *t*-test. (*, *P* < 0.05; **, *P* < 0.01).

**Additional file 4: Fig. S4. **Phenotype of *TLR3*-OE Arabidopsis lines. Different leaves from *TLR3*-OE lines and Col-0 are shown.

**Additional file 5: Fig. S5.** Secondary branches number in *TLR3*-OE lines and Col-0. *TLR3*-OE lines show fewer secondary branches than Col-0.

**Additional file 6: Fig. S6.** Biomass of *TLR3*-OE lines and Col-0. Data are means SD (*n* = 3). Asterisks indicate significant differences between *ArabidopsisTLR3*-OE lines and Col-0 by Student’s *t*-test. (***, *P* < 0.001).

**Additional file 7: Fig. S7. **The TLR3 gene was divided into three segments: full-length amino acids, 1-111 amino acids and 112-206 amino acids, and the self-activation detection of the three-segment gene.

**Additional file 8: Fig. S8.** Phylogenetic relationship of ECT2 proteins. The reconstruction of the phylogenetic tree was performed by the neighbor-joining method in MEGA 7, using the amino acid sequence as input. The corresponding gene IDs are listed as follows: AaECT2: PWA54528, TcECT2: GEX60941, CcECT3: XP_024985184, AlECT2: KAI3706292, EcECT2: XP_043630326, AlECT3: KAI3706293, CcECT2: XP_024985182, HaECT4: XP_021972327, AtECT2: AT3G13460.

**Additional file 9: Fig. S9.** Appearance of glandular and non-glandular trichomes on the surface of *TLR3*-OE *A. annua *leave petioles.

**Additional file 10: Fig. S10. **Size of glandular and non-glandular trichomes on the surface of *TLR3*-OE *A. annua *leaves. Data are means SD (*n* = 3). Asterisks indicate significant differences between *TLR3*-OE lines and WT by Student’s *t*-test. (***, *P* < 0.001).

**Additional file 11: Fig. S11. **Cell size in *TLR3*-OE *A. annua* leaves. Data are means SD (*n* = 3). Asterisks indicate significant differences between *TLR3*-OE *Arabidopsis* lines and Col-0 by Student’s *t*-test. (***, *P* < 0.001).

**Additional file 12: Fig. S12. **Contents of long-chain fatty acids in *TLR3*-OE *A. annua *plants. The contents of long-chain fatty acids were measured by LC–MS/MS. LDGTS, lyso-diacylglyceryltrimethylhomoserine; ADGGA, acyl diacylglycerol glucuronic acid; DGDG, disaccharide diglycerides; ADGGA, acyl diacylglycerol glucuronic acid; FFA, free fatty acids; DG, diglyceride; MGDG, monosaccharide diglycerides; PA, phosphatidic acid; DGTS, diacylglyceryltrimethylhoserine; Cer, ceramide; PC, phosphatidylcholine; PE, phosphatidylethanolamine; PI, phosphatidylinositol; PS, phosphatidylserine; TG, triglyceride; PG, phosphatidylglycerol. Green indicates low abundance; red indicates high abundance.

**Additional file 13: Fig. S13. **Summarized expression profiles of genes from the TCA cycle in *A. annua* overexpressing *TLR3*. The expression of pyruvate kinase (*PK1*), pyruvate kinase 2-like (*PK2L*), *PK3*, *PK4* and isocitrate dehydrogenase (*IDH*) is significantly increased in the transgenic plants, while the expression of *PK2*, succinate dehydrogenase (*SDH*), citrate synthase (*CS*) and phosphoenolpyruvate carboxylase (*PEPC*) was significantly downregulated in the transgenic plants.

**Additional file 14: Table S1. **Primers used in this study.

**Additional file 15: Table S2. **Six MYB TFs involved in trichome development by GO analysis.

**Additional file 16: Table S3. **Summary of the transcriptome analysis.

**Additional file 17: Table S4.** Significantly expressed genes

**Additional file 18: Table S5.** Seven genes involved in lipid biosynthesis in the top 500 most significantly expressed genes.

## Data Availability

The datasets used and/or analysed during the current study are available from the corresponding author on reasonable request.

## Abbreviations

TLR3: TrichomeLess Regulator 3
ACT: Artemisinin-based combination therapy
FPP: Farnesyl diphosphate
DHAA: Dihydroartemisinic acid
ADS: Amorpha-4,11-diene synthase
CYP71AV1: Amorphadiene 12-hydroxylase
DBR2: Artemisinic aldehydeΔ11(13) reductase
ALDH1: Aldehyde dehydrogenase 1
TFs: Transcription factors
ETC1: EVOLUTIONARILY CONSERVED C-TERMINAL REGION 1
TRY: Triptychon
bHLH: Basic helix-loop-helix
TTG1: TRANSPARENT TESTA GLABRA1
CPC: CAPRICE
HD: Homeodomain
ZIP: Leucine zipper
TLR1: TrichomeLess Regulator 1
WOX1: WUSCHEL-RELATED HOMEOBOX 1
RNAi: RNA interference
CycTL: Cyclin trichomeless
YFP: Yellow fluorescent protein
GUS: ß-glucuronidase
OE: Overexpressing
TLR3i: TLR3-intron
WER: WEREWOLF
ZHD8: ZINC FINGER HOMEODOMAIN 8
SCN1: SUPERCENTIPEDE 1
NFY: NUCLEAR FACTOR Y
Y2H: Yeast two-hybrid
GST: Glutathione S-transferase
RNA EMSA: RNA electrophoretic mobility shift assay
RNAi: MRNA knockdown
Cas9: (CRISPR)/CRISPR-associated nuclease 9
TB: Toluidine blue
SUR2: Sphingolipid C4 hydroxylase
PEARLI 4: Phospholipase-like protein
LTP: Lipid transfer protein
DIR1: Bifunctional inhibitor/plant lipid transfer protein/seed storage helical domain-containing protein
LDGTS: Lyso-diacylglyceryltrimethylhomoserine
FFA: Free fatty acids
DGDG: Disaccharide diglycerides
DG: Diglyceride
ADGGA: Acyl diacylglycerol glucuronic acid
MGDG: Monosaccharide diglycerides
PA: Phosphatidic acid
DGTS: Diacylglyceryltrimethylhoserine
Cer: Ceramide
PC: Phosphatidylcholine
PE: Phosphatidylethanolamine
PI: Phosphatidylinositol
PS: Phosphatidylserine
TG: Triglyceride
PG: Phosphatidylglycerol
TCA: Tricarboxylic acid
PK2: Pyruvate kinase 2
SDH: Succinate dehydrogenase subunit 4
CS: Citrate synthase
PEPC: Phosphoenolpyruvate carboxylase
VLCFA: Very-long-chain fatty acid
NaClO: Sodium hypochlorite
MS: Murashige and Skoog
6-BA: 6-Benzylaminopurine
NAA: Naphthaleneacetic acid
ART: Arteminisin
AA: Artemisinic acid
LC–MS/M: Liquid chromatography-tandem mass spectrometry
MTBE: Methyl tert-butyl ether
PK1: Pyruvate kinase
PK2L: Pyruvate kinase 2-like
IDH: Isocitrate dehydrogenase
