# Supplementary figures and images for: TrichomeLess Regulator 3 is required for trichome initial and cuticle biosynthesis in Artemisia annua
Source: Mol Hortic. 2024 Mar 19;4:10. doi: 10.1186/s43897-024-00085-4 (PMC10949617; doi:10.1186/s43897-024-00085-4)

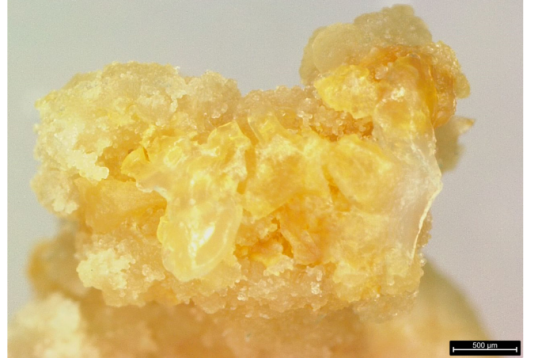


**Fig. S1.** Control calli cannot be stained.

Supplement: Supplementary file 1 — Additional file 1: Fig. S1. Control calli cannot be stained. [file 43897_2024_85_MOESM1_ESM.docx]

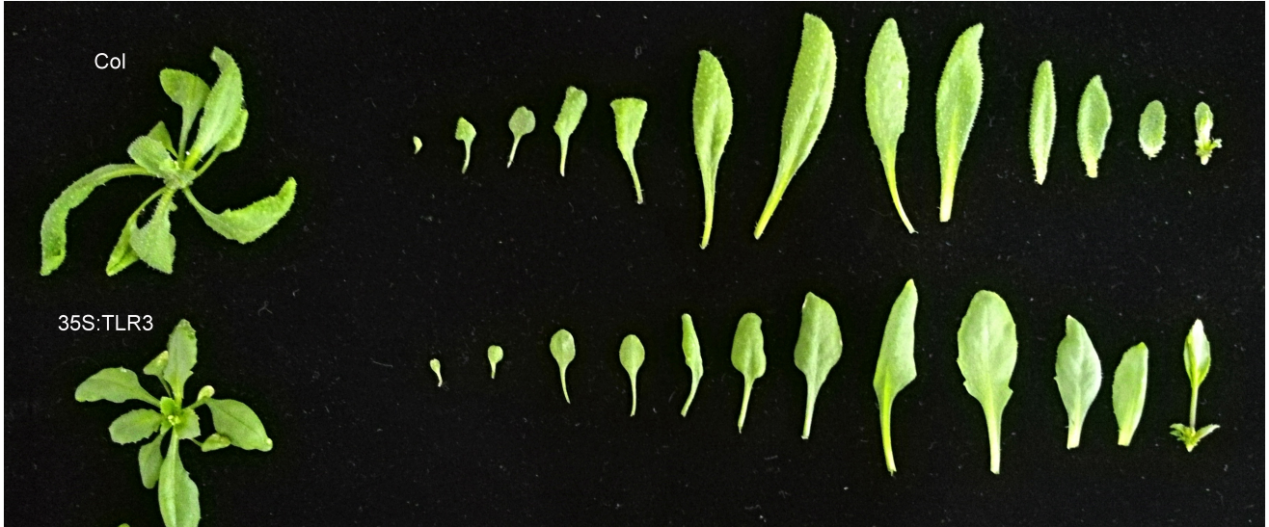


**Fig. S4.** Phenotype of *TLR3*-OE Arabidopsis lines. Different leaves from *TLR3*-OE lines and Col-0 are shown.

Supplement: Supplementary file 4 — Additional file 4: Fig. S4. Phenotype of TLR3-OE Arabidopsis lines. Different leaves from TLR3-OE lines and Col-0 are shown. [file 43897_2024_85_MOESM4_ESM.docx]
