# Supplementary material for: TrichomeLess Regulator 3 is required for trichome initial and cuticle biosynthesis in Artemisia annua
Source: Mol Hortic. 2024 Mar 19;4:10. doi: 10.1186/s43897-024-00085-4 (PMC10949617; doi:10.1186/s43897-024-00085-4)
Supplement: Supplementary file 2 — Additional file 2: Fig. S2. Diagram of the TLR3 locus and phenotype of TLR3-OE and TLR3-intron-OE lines. (a) The coding sequence of TLR3 is 621 bp in length; the length of the TLR3-intron is 834 bp. (b) Analysis of TLR3 gene amplification by agarose gel electrophoresis. (c) TLR3-intron-OE lines have fewer trichomes than TLR3-OE plants. [file 43897_2024_85_MOESM2_ESM.docx]

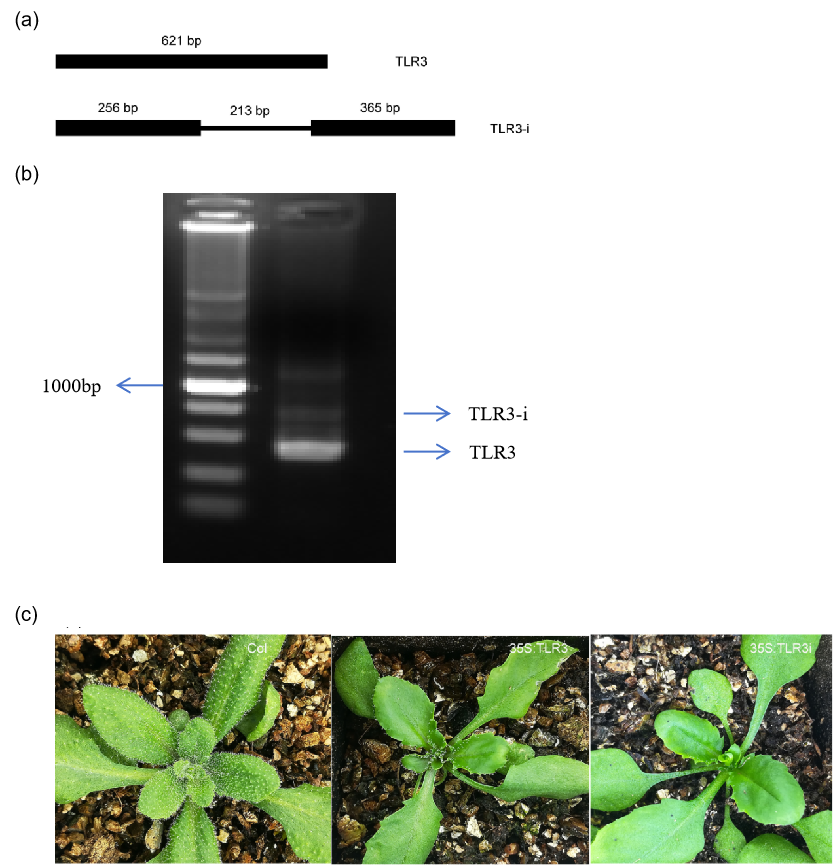


**Fig. S2.** Diagram of the *TLR3* locus and phenotype of *TLR3*-OE and *TLR3*-intron-OE lines. (a) The coding sequence of *TLR3* is 621 bp in length; the length of the *TLR3*-intron is 834 bp. (b) Analysis of *TLR3* gene amplification by agarose gel electrophoresis. (c) TLR3-intron-OE lines have fewer trichomes than *TLR3*-OE plants.
