# Supplementary material for: TrichomeLess Regulator 3 is required for trichome initial and cuticle biosynthesis in Artemisia annua
Source: Mol Hortic. 2024 Mar 19;4:10. doi: 10.1186/s43897-024-00085-4 (PMC10949617; doi:10.1186/s43897-024-00085-4)
Supplement: Supplementary file 3 — Additional file 3: Fig. S3. Genes involved in trichome development and root hair development expression levels in TLR3-OE Arabidopsis lines. Data are means SD (n = 3). Asterisks indicate significant differences between TLR3-OE lines and Col-0 by Student’s t-test. (*, P < 0.05; **, P < 0.01). [file 43897_2024_85_MOESM3_ESM.docx]

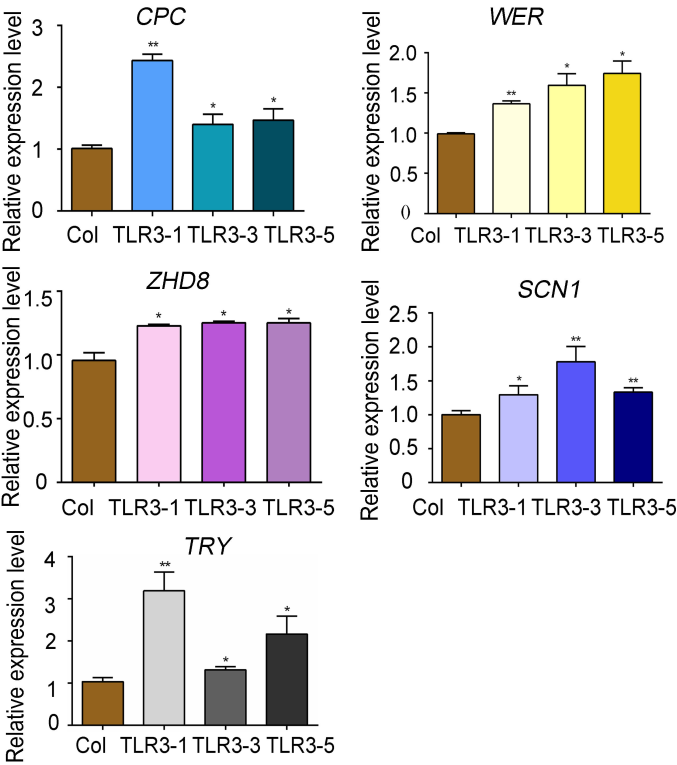


**Fig. S3.** Genes involved in trichome development and root hair development expression levels in *TLR3*-OE Arabidopsis lines. Data are means SD (*n* = 3). Asterisks indicate significant differences between *TLR3*-OE lines and Col-0 by Student’s *t*-test. (*, *P* < 0.05; **, *P* < 0.01.)
