# Supplementary material for: TrichomeLess Regulator 3 is required for trichome initial and cuticle biosynthesis in Artemisia annua
Source: Mol Hortic. 2024 Mar 19;4:10. doi: 10.1186/s43897-024-00085-4 (PMC10949617; doi:10.1186/s43897-024-00085-4)
Supplement: Supplementary file 5 — Additional file 5: Fig. S5. Secondary branches number in TLR3-OE lines and Col-0. TLR3-OE lines show fewer secondary branches than Col-0. [file 43897_2024_85_MOESM5_ESM.docx]

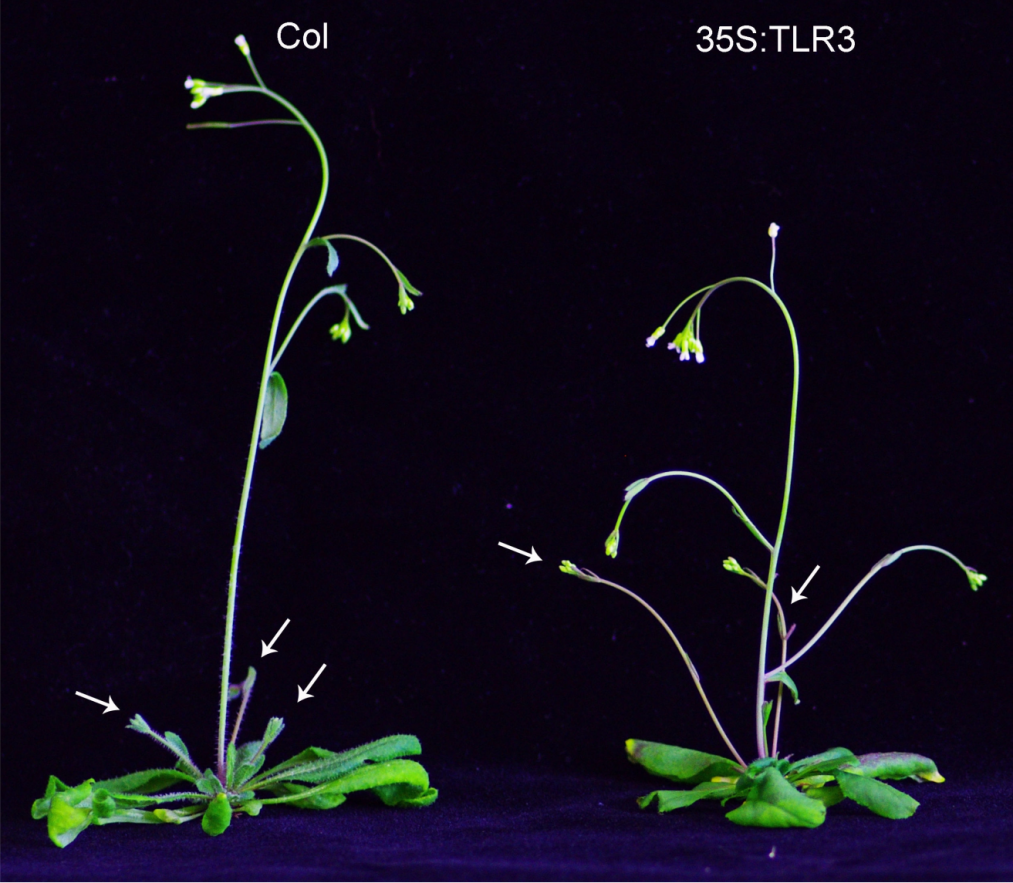


**Fig. S5.** Secondary branches number in *TLR3*-OE lines and Col-0. *TLR3*-OE lines show fewer secondary branches than Col-0.
