# Supplementary material for: TrichomeLess Regulator 3 is required for trichome initial and cuticle biosynthesis in Artemisia annua
Source: Mol Hortic. 2024 Mar 19;4:10. doi: 10.1186/s43897-024-00085-4 (PMC10949617; doi:10.1186/s43897-024-00085-4)
Supplement: Supplementary file 6 — Additional file 6: Fig. S6. Biomass of TLR3-OE lines and Col-0. Data are means SD (n = 3). Asterisks indicate significant differences between ArabidopsisTLR3-OE lines and Col-0 by Student’s t-test. (***, P < 0.001). [file 43897_2024_85_MOESM6_ESM.docx]

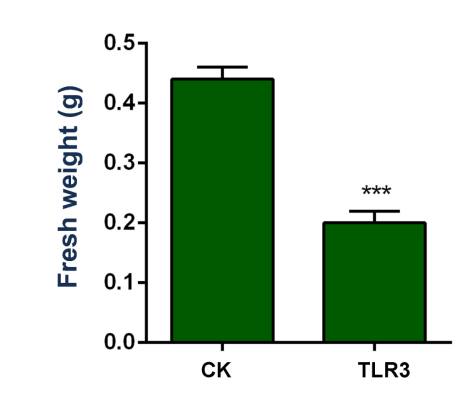


**Fig. S6.** Biomass of *TLR3*-OE lines and Col-0. Data are means SD (*n* = 3). Asterisks indicate significant differences between Arabidopsis *TLR3*-OE lines and Col-0 by Student’s *t*-test. (***, *P* < 0.001.)
