# Supplementary material for: TrichomeLess Regulator 3 is required for trichome initial and cuticle biosynthesis in Artemisia annua
Source: Mol Hortic. 2024 Mar 19;4:10. doi: 10.1186/s43897-024-00085-4 (PMC10949617; doi:10.1186/s43897-024-00085-4)
Supplement: Supplementary file 7 — Additional file 7: Fig. S7. The TLR3 gene was divided into three segments: full-length amino acids, 1-111 amino acids and 112-206 amino acids, and the self-activation detection of the three-segment gene. [file 43897_2024_85_MOESM7_ESM.docx]

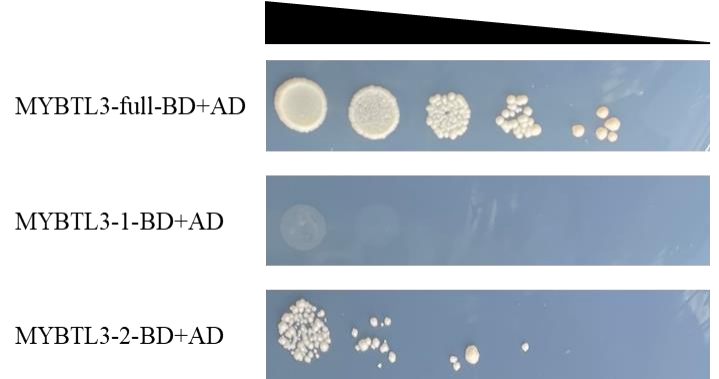


ΔTLR3’-BD+AD

ΔTLR3-BD+AD

TLR3-full-BD+AD

**Fig S7.** The *TLR3* gene was divided into three segments: full-length amino acids, 1-111 amino acids and 112-206 amino acids, and the self-activation detection of the three-segment gene.
