# Supplementary material for: TrichomeLess Regulator 3 is required for trichome initial and cuticle biosynthesis in Artemisia annua
Source: Mol Hortic. 2024 Mar 19;4:10. doi: 10.1186/s43897-024-00085-4 (PMC10949617; doi:10.1186/s43897-024-00085-4)
Supplement: Supplementary file 8 — Additional file 8: Fig. S8. Phylogenetic relationship of ECT2 proteins. The reconstruction of the phylogenetic tree was performed by the neighbor-joining method in MEGA 7, using the amino acid sequence as input. The corresponding gene IDs are listed as follows: AaECT2: PWA54528, TcECT2: GEX60941, CcECT3: XP_024985184, AlECT2: KAI3706292, EcECT2: XP_043630326, AlECT3: KAI3706293, CcECT2: XP_024985182, HaECT4: XP_021972327, AtECT2: AT3G13460. [file 43897_2024_85_MOESM8_ESM.docx]

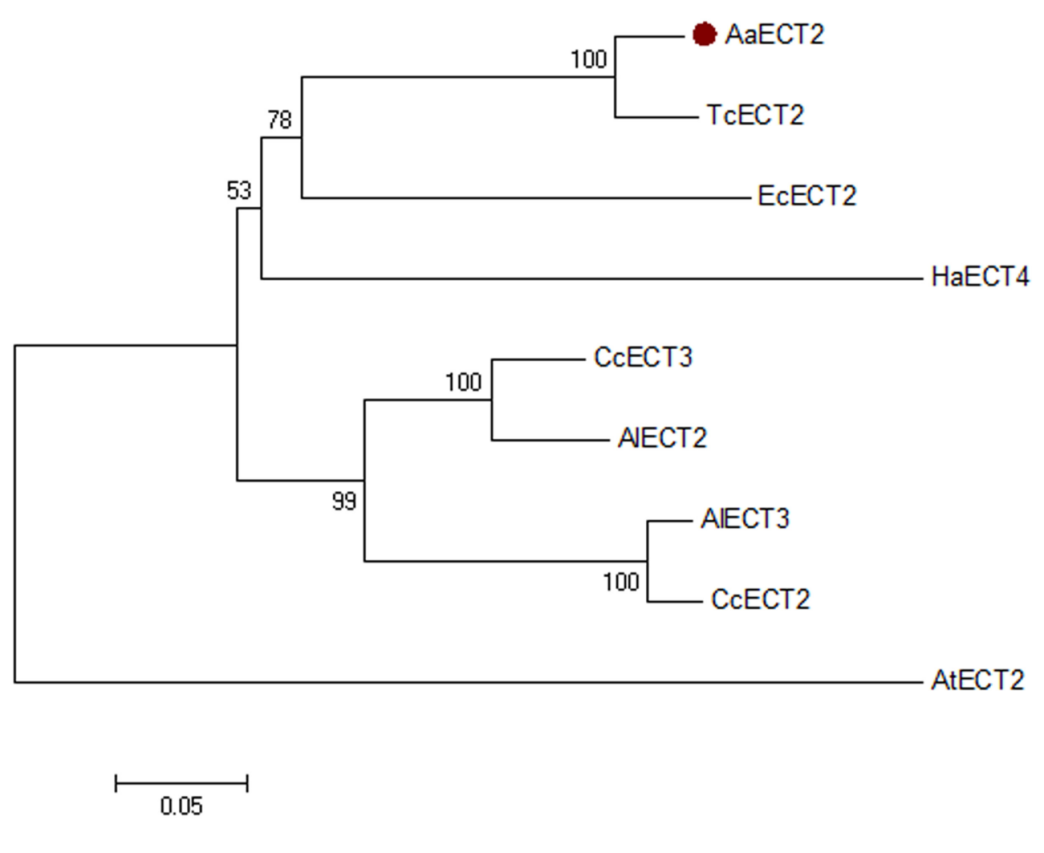


**Fig. S8.** Phylogenetic relationship of ECT2 proteins. The reconstruction of the phylogenetic tree was performed by the neighbor-joining method in MEGA 7, using the amino acid sequence as input. The corresponding gene IDs are listed as follows: AaECT2: PWA54528, TcECT2: GEX60941, CcECT3: XP_024985184, AlECT2: KAI3706292, EcECT2: XP_043630326, AlECT3: KAI3706293, CcECT2: XP_024985182, HaECT4: XP_021972327, AtECT2: AT3G13460.
