# Supplementary material for: TrichomeLess Regulator 3 is required for trichome initial and cuticle biosynthesis in Artemisia annua
Source: Mol Hortic. 2024 Mar 19;4:10. doi: 10.1186/s43897-024-00085-4 (PMC10949617; doi:10.1186/s43897-024-00085-4)
Supplement: Supplementary file 9 — Additional file 9: Fig. S9. Appearance of glandular and non-glandular trichomes on the surface of TLR3-OE A. annua leave petioles. [file 43897_2024_85_MOESM9_ESM.docx]

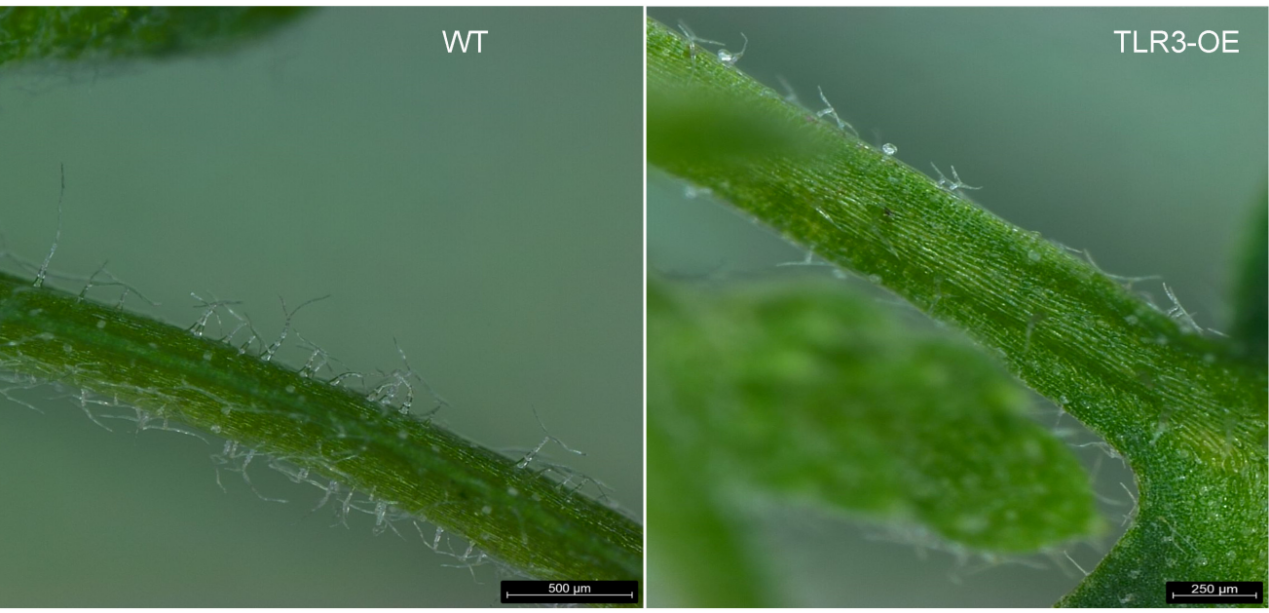


**Fig. S9.** Appearance of glandular and non-glandular trichomes on the surface of *TLR3*-OE *A. annua* leave petioles.
