# Supplementary material for: TrichomeLess Regulator 3 is required for trichome initial and cuticle biosynthesis in Artemisia annua
Source: Mol Hortic. 2024 Mar 19;4:10. doi: 10.1186/s43897-024-00085-4 (PMC10949617; doi:10.1186/s43897-024-00085-4)
Supplement: Supplementary file 11 — Additional file 11: Fig. S11. Cell size in TLR3-OE A. annua leaves. Data are means SD (n = 3). Asterisks indicate significant differences between TLR3-OE Arabidopsis lines and Col-0 by Student’s t-test. (***, P < 0.001). [file 43897_2024_85_MOESM11_ESM.docx]

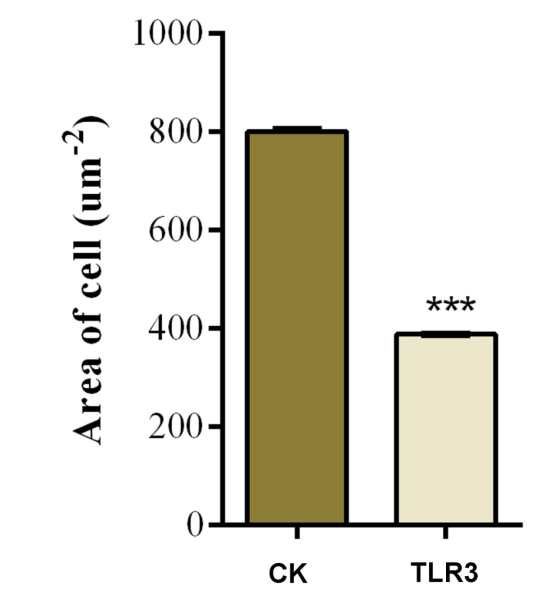


**Fig. S11.** Cell size in *TLR3*-OE *A. annua* leaves. Data are means SD (*n* = 3). Asterisks indicate significant differences between *TLR3*-OE Arabidopsis lines and Col-0 by Student’s *t*-test. (***, *P* < 0.001).
