# Supplementary material for: TrichomeLess Regulator 3 is required for trichome initial and cuticle biosynthesis in Artemisia annua
Source: Mol Hortic. 2024 Mar 19;4:10. doi: 10.1186/s43897-024-00085-4 (PMC10949617; doi:10.1186/s43897-024-00085-4)
Supplement: Supplementary file 12 — Additional file 12: Fig. S12. Contents of long-chain fatty acids in TLR3-OE A. annua plants. The contents of long-chain fatty acids were measured by LC–MS/MS. LDGTS, lyso-diacylglyceryltrimethylhomoserine; ADGGA, acyl diacylglycerol glucuronic acid; DGDG, disaccharide diglycerides; ADGGA, acyl diacylglycerol glucuronic acid; FFA, free fatty acids; DG, diglyceride; MGDG, monosaccharide diglycerides; PA, phosphatidic acid; DGTS, diacylglyceryltrimethylhoserine; Cer, ceramide; PC, phosphatidylcholine; PE, phosphatidylethanolamine; PI, phosphatidylinositol; PS, phosphatidylserine; TG, triglyceride; PG, phosphatidylglycerol. Green indicates low abundance; red indicates high abundance. [file 43897_2024_85_MOESM12_ESM.docx]

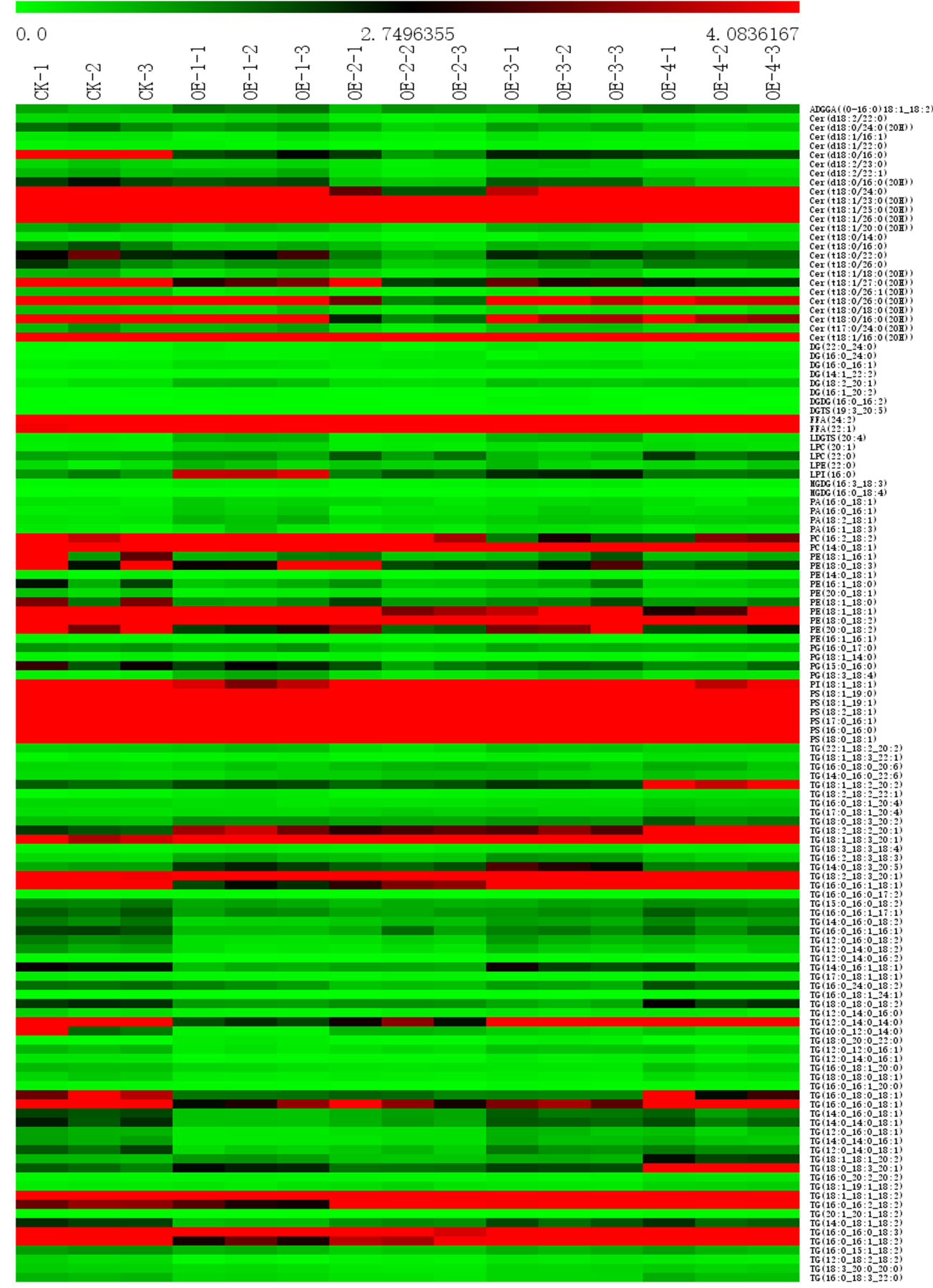


**Fig. S12.** Contents of long-chain fatty acids in *TLR3*-OE *A. annua* plants. The contents of long-chain fatty acids were measured by LC–MS/MS. LDGTS, lyso-diacylglyceryltrimethylhomoserine; ADGGA, acyl diacylglycerol glucuronic acid; DGDG, disaccharide diglycerides; ADGGA, acyl diacylglycerol glucuronic acid; FFA, free fatty acids; DG, diglyceride; MGDG, monosaccharide diglycerides; PA, phosphatidic acid; DGTS, diacylglyceryltrimethylhoserine; Cer, ceramide; PC, phosphatidylcholine; PE, phosphatidylethanolamine; PI, phosphatidylinositol; PS, phosphatidylserine; TG, triglyceride; PG, phosphatidylglycerol. Green indicates low abundance; red indicates high abundance.
