# Supplementary material for: TrichomeLess Regulator 3 is required for trichome initial and cuticle biosynthesis in Artemisia annua
Source: Mol Hortic. 2024 Mar 19;4:10. doi: 10.1186/s43897-024-00085-4 (PMC10949617; doi:10.1186/s43897-024-00085-4)
Supplement: Supplementary file 13 — Additional file 13: Fig. S13. Summarized expression profiles of genes from the TCA cycle in A. annua overexpressing TLR3. The expression of pyruvate kinase (PK1), pyruvate kinase 2-like (PK2L), PK3, PK4 and isocitrate dehydrogenase (IDH) is significantly increased in the transgenic plants, while the expression of PK2, succinate dehydrogenase (SDH), citrate synthase (CS) and phosphoenolpyruvate carboxylase (PEPC) was significantly downregulated in the transgenic plants. [file 43897_2024_85_MOESM13_ESM.docx]

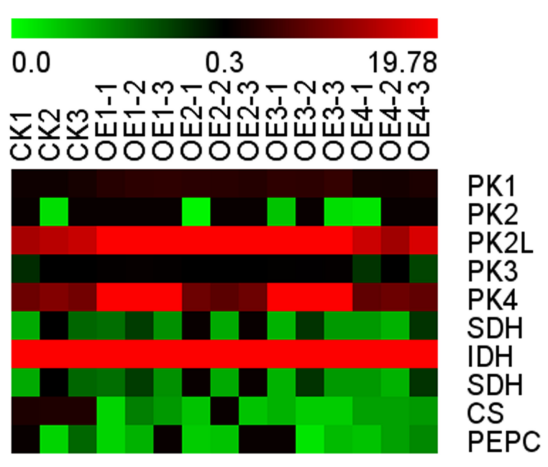


**Fig. S13.** Summarized expression profiles of genes from the TCA cycle in *A. annua* overexpressing *TLR3*. The expression of pyruvate kinase (*PK1*), pyruvate kinase 2-like (*PK2L*), *PK3*, *PK4* and isocitrate dehydrogenase (*IDH*) is significantly increased in the transgenic plants, while the expression of *PK2*, succinate dehydrogenase (*SDH*), citrate synthase (*CS*) and phosphoenolpyruvate carboxylase (*PEPC*) was significantly downregulated in the transgenic plants.
