# Supplementary material for: TrichomeLess Regulator 3 is required for trichome initial and cuticle biosynthesis in Artemisia annua
Source: Mol Hortic. 2024 Mar 19;4:10. doi: 10.1186/s43897-024-00085-4 (PMC10949617; doi:10.1186/s43897-024-00085-4)
Supplement: Supplementary file 14 — Additional file 14: Table S1. Primers used in this study. [file 43897_2024_85_MOESM14_ESM.docx]

Primers used in this assay

| Name | Sequence（5’-3’） |
| --- | --- |
| *MYBTL3*-F | ATGAGGAATGTGTTACCGGC |
| *MYBTL3*-R | TAGAGCAGTTGGGGTGCGTT |
| pJET1.2-R | AAGAACATCGATTTTCCATGGCAG |
| PHB-*MYBTL3*-F | CGGGCCATGAATTCCTGCAG ATGAGGAATGTGTTACCGGC |
| PHB-*MYBTL3*-R | GCTCTAGAACTAGTGGATCC TAGAGCAGTTGGGGTGCGTT |
| RBC48A | GCATTGAACTTGACGAACGTTGTCGA |
| YFP-*MYBTL3*-F | CAGTCTCTCTCTCCAAGCTT ATGAGGAATGTGTTACCGGC |
| YFP-*MYBTL3*-R | CTCACCATACTAGTGAGCTC  TAGAGCAGTTGGGGTGCGTT |
| *proMYBTL3*-F | GCTCTAATAAGCAAATCCAAAAC |
| *proMYBTL3*-F | TTCTAAAACACAAAAATGCCCGC |
| 1391-*proMYBTL3*-F | GGCTGCAGGTCGACGGATCC  GCTCTAATAAGCAAATCCAAAAC |
| 1391-*proMYBTL3*-R | GTGGACTCCTCTTAGAATTC  TTCTAAAACACAAAAATGCCCGC |
| M13F | TGTAAAACGACGGCCAGT |
| pENTR-*MYBTL3*-F | CACCCTCCGCCGATGAAGAAGACCT |
| pENTR-*MYBTL3*-R | TCCGGTGAAGAAGATGAATGG |
| pENTR-F | CTACAAACTCTTCCTGTTAGTTAG |
| P27-3 | GAGCTACACATGCTCAGG |
| P27-5 | GGGATGACGCACAATCC |
| sgRNA-*MYBTL3*-F | GATTAACTCCATCAACCCGCACAG |
| sgRNA-*MYBTL3*-R | AAACCTGTGCGGGTTGATGGAGTT |
| BD-*MYBTL3*-full-F | TGGCCATGGAGGCCGAATTC  ATGAGGAATGTGTTACCGGC |
| BD-*MYBTL3*-full-R | CGCTGCAGGTCGACGGATCC  TAGAGCAGTTGGGGTGCGTTC |
| BD-*MYBTL3*-1-F | TGGCCATGGAGGCCGAATTC  ATGAGGAATGTGTTACCGGC |
| BD-*MYBTL3*-1-R | CGCTGCAGGTCGACGGATCC  CTTGCTAAGGTGAGTATTCC |
| M13F | GTAAAACGACGGCCAGT |
| BD-*MYBTL3*-2-F | TGGCCATGGAGGCCGAATTC  AAGTTAACAAGCCAAGGGAT |
| BD-*MYBTL3*-2-R | CGCTGCAGGTCGACGGATCC  TAGAGCAGTTGGGGTGCGTTC |
| 3’BD | TAAGAGTCACTTTAAAATTTGTATC |
| pGEX4T-1-*MYBTL3*-F | CGCGTGGATCCCCGGAATTC  ATGAGGAATGTGTTACCGGC |
| pGEX4T-1-*MYBTL3*-R | TCACGATGCGGCCGCTCGAG  TAGAGCAGTTGGGGTGCGTT |
| pGEX3' | CCGGGAGCTGCATGTGTCAGAGG |
| *CYCTL*-F | ATGAGCCGAAGAAACGGTGG |
| *CYCTL*-R | ACTCTTTTTTGTTTTCTTGAG |
| AD-*CYCTL*-F | CCATGGAGGCCAGTGAATTC  ATGAGCCGAAGAAACGGTGG |
| AD-*CYCTL*-R | AGCTCGAGCTCGATGGATCC  ACTCTTTTTTGTTTTCTTGAG |
| 3’AD | AGATGGTGCACGATGCACAG |
| pET32a*-CYCTL*-F | GGATCCGAATTCGAGCTC  ATGAGCCGAAGAAACGGTGG |
| pET32a-*CYCTL*-R | GAGTGCGGCCGCAAGCTT  ACTCTTTTTTGTTTTCTTGAG |
| YFP*-CYCTL*-F | CAGTCTCTCTCTCCAAGCTT ATGAGCCGAAGAAACGGTGG |
| YFP*-CYCTL*-R | CTCACCATACTAGTGAGCTC  ACTCTTTTTTGTTTTCTTGAG |
| PHB*-CYCTL*-F | CGGGCCATGAATTCCTGCAG ATGAGCCGAAGAAACGGTGG |
| PHB*-CYCTL*-R | GCTCTAGAACTAGTGGATCC ACTCTTTTTTGTTTTCTTGAG |
| pBiFC-104C-*CYCTL*-F | TACAATTACAGGTACCCGGG  ATGAGCCGAAGAAACGGTGG |
| pBiFC-104C-*CYCTL*-R | CACCGCCGTCGACTCTAGA  ACTCTTTTTTGTTTTCTTGAG |
| pBiFC-104C-R | CTTCTCGTTGGGGTCTTTGCT |
| *ZWI*-F | ATGTATTTATGTGCATCGTGCATG |
| *ZWI*-R | AAAAAACACAGCTGTATTGCTGCTG |
| AD-*ZWI*-F | CCATGGAGGCCAGTGAATTC  ATGTATTTATGTGCATCGTGCATG |
| AD-*ZWI*-R | AGCTCGAGCTCGATGGATCC  AAAAAACACAGCTGTATTGCTGCTG |
| pET32a*-ZWI*-F | GGATCCGAATTCGAGCTC  ATGTATTTATGTGCATCGTGCATG |
| pET32a*-ZWI*-R | GAGTGCGGCCGCAAGCTT  AAAAAACACAGCTGTATTGCTGCTG |
| *NF-Y*-F | ATGGATCAGCAAGGGCATGG |
| *NF-Y*-R | TTAAGCATCTTCCTGTGGTTCTTG |
| AD-*NF-Y*-F | CCATGGAGGCCAGTGAATTC  ATGGATCAGCAAGGGCATGG |
| AD-*NF-Y*-R | AGCTCGAGCTCGATGGATCC  TTAAGCATCTTCCTGTGGTTCTTG |
| pET32a*-NFY*-F | GGATCCGAATTCGAGCTC  ATGGATCAGCAAGGGCATGG |
| pET32a*-NFY*-R | GAGTGCGGCCGCAAGCTT  AGCATCTTCCTGTGGTTCTTGTTG |
| T7t | GCTAGTTATTGCTCAGCGG |
| *TAR1*-F | ATGGGTCAAAAGAAGTTTAGG |
| *TAR1*-R | CTAATTCGTATTAAGCAATTCTTC |
| BD-*TAR1*-F | TGGCCATGGAGGCCGAATTC ATGGGTCAAAAGAAGTTTAGG |
| BD-*TAR1*-F | CGCTGCAGGTCGACGGATCC CTAATTCGTATTAAGCAATTCTTC |
| pBiFC-106N-*TAR1*-F | CGAGGACGCCGGCGGATCC  ATGGGTCAAAAGAAGTTTAGG |
| pBiFC-106N-*TAR1*-R | AGGTCGACTCTAGAGGATCC  ATTCGTATTAAGCAATTCTTCAATC |
| pBiFC-106N-F | GCGACACCCTGGTGAACCG |
| *ECT2*-F | ATGGCTGCTGTTGTTCCTTC |
| *ECT2*-R | GCAACCATTTGCAATCCCATTTG |
| pET32a-*ECT2*-F | GGATCCGAATTCGAGCTC  ATGGCTGCTGTTGTTCCTTC |
| pET32a-*ECT2*-R | GAGTGCGGCCGCAAGCTT  GCAACCATTTGCAATCCCATTTG |
| AT-actin-QF | GCATGAAGATCAAGGTGGTTGCAC |
| AT-actin-QR | ATGGACCTGACTCATCGTACTCACT |
| ATGL2-QF | GAGCCCACGGGAGTCATTGTCT |
| ATGL2-QF | TGCCACTGAGTTGCCTCTGTCT |
| ATTTG1-QF | CTCCTTCTCTCCGTCGTCCTTC |
| ATTTG1-QR | CGGCTCTACATCGTTCCAATCG |
| ATTTG2-QF | TCGCAAGGCTCAGTGGAATCAG |
| ATTTG2-QR | TGGATCATCACTCGCTCGTTCA |
| ATTRY-QF | GACTTGTCGGTGATAGGTGGGA |
| ATTRY-QR | GGACGGTGAGGCTTGGTATGTT |
| ATCPC-QF | ACGACGACGGAGACAGAGCAA |
| ATCPC-QR | GATCCTTCCGGCGATCAACTCC |
| ATETC1-QF | GCAGCGTAAGTCGAAGCATCTT |
| ATETC1-QR | CTGCTGTTCTTCCTGGAATCCT |
| ATSCN1-QF | CGAGGAGGAAGAAGACGACGAT |
| ATSCN1-QR | CAGGAGATATGATGGCGAGGCT |
| ATWER-QF | TGGCAAAGGTCACTGGAATCGT |
| ATWER-QR | ACCAAGCAACTTGTGGAGCCTA |
| ATKIS-QF | GCGGCTAAGACTGCTGATATGA |
| ATKIS-QR | TCCTTCTCATCCGTCTCTTCCA |
| ATERH3-QF | GCTCTCAGGAGGAGGTTGGAA |
| ATERH3-QR | CGCTATCTTACGCCGCATCC |
| ATZHD8-QF | GCGGTTGCGGTTGCGACTTA |
| ATZHD8-QR | AGGAAGCTGGTGACGGTTGTGA |
| ATMYB106-QF | GGCGGGCGTGGGGAATGA |
| ATMYB106-QR | TCGCGGAATCGGACGGTGAA |
| QAA-actin-F | CCAGGCTGTTCAGTCTCTGTAT |
| QAA-actin-R | CGCTCGGTAAGGATCTTCATCA |
| QAAADS-F | AATGGGCAAATGAGGGACAC |
| QAAADS-R | TTTCAAGGCTCGATGAACTATG |
| QAACYP71AV1-F | TCATTTCAGTCGCTT |
| QAACYP71AV1-R | CCAGTTTGCCTCAGTA |
| QAADBR2-F | ACTGCTGGTGGCTTTCTTA |
| QAADBR2-R | ACCCTCGACTTGTTCCTTA |
| QAAALDH1-F | GGACTTGCCTCAGGTGTAT |
| QAAALDH1-R | GTGCCTCTAATCCTTGTTC |
| QAAMYBTL3-F | CGCACAGTGGTCCTCGATCTGA |
| QAAMYBTL3-R | TCGCTGCACATTTCGTCGTTGA |
| QAAMIXTA1-F | GGTGGTCAGCAATAGCAACTCA |
| QAAMIXTA1-R | TTGGCTTGTGGCTCACTGGAT |
| QAATAR1-F | gggttcttgggtttccgaga |
| QAATAR1-R | gctgttgtagatgttggtgg |
| QAAZWI-F | TGAAGGCTGGACCACGACATAC |
| QAAZWI-R | TCCTCAACAGCATTAGCAACGG |
| QAACYCTL-F | GCTCGTGGACCGATGTCACAAT |
| QAACYCTL-R | GGGCACCCGACTAGCATCATAG |
| QAAGSW1-F | TCTCGTCAAAGACACACACATTC |
| QAAGSW1-R | TTGTTCGTAGTTGCTGTAGTGCT |
| QAAGSW2-F | TTCCTCATCTTCAACTTCACCAC |
| QAAGSW2-R | CGCTACTCGTGGGAGAAACAT |
| QAAHD8-F | AAATGCTAGATTGAAGGACGAA |
| QAAHD8-R | AAGATGGTACAACCGATGAGG |
