# Supplementary material for: TrichomeLess Regulator 3 is required for trichome initial and cuticle biosynthesis in Artemisia annua
Source: Mol Hortic. 2024 Mar 19;4:10. doi: 10.1186/s43897-024-00085-4 (PMC10949617; doi:10.1186/s43897-024-00085-4)
Supplement: Supplementary file 15 — Additional file 15: Table S2. Six MYB TFs involved in trichome development by GO analysis. [file 43897_2024_85_MOESM15_ESM.docx]

Table S2 Six MYB TFs involved in trichome development by GO analysis

| Function | biological processes | Gene name |
| --- | --- | --- |
| trichomes differentiated | GO:0010026 | AA006100 |
| trichomes branching | GO:0010091 | AA366500 AA300940 AA0737504 |
| trichomes initial | GO:0010090 | AA005270 AA006100 AA179640 |
